# pH-Dependent Vibrational Dynamics Drives Excited-State Quenching in the Phycobiliprotein Complex PC645

**DOI:** 10.1021/jacs.6c07743

**Published:** 2026-07-03

**Authors:** Sayan Maity, Yue Hu, Dongyu Lyu, Jingyun Wu, Coral O’Brien, Michael H. Hecht, Leah C. Spangler, Gregory D. Scholes, Ulrich Kleinekathöfer

**Affiliations:** † School of Science, 84498 Constructor University, Campus Ring 1, 28759 Bremen, Germany; ‡ Department of Physics and Astronomy and Thomas Young Centre, 4919 University College London, London WC1E 6BT, U.K; § Department of Chemistry, 6740 Princeton University, Princeton, New Jersey 08544, United States; ∥ Center for Pharmaceutical Engineering and Sciences, 6889 Virginia Commonwealth University, Richmond, Virginia 23284, United States; ⊥ Department of Chemical and Life Science Engineering, 6889 Virginia Commonwealth University, Richmond, Virginia 23284, United States

## Abstract

Phycocyanin 645 (PC645) is a closed-form light-harvesting complex found in the lumen of the photosynthetic membrane of cryptophyte algae. These peripheral antenna complexes contain bilin chromophores that absorb sunlight and transfer excitation energy to the core antenna complexes embedded in the thylakoid membrane. The location of cryptophyte antenna complex on the luminal side of the membrane is unusual. During photosynthetic activity, the pH of the lumen drops, by up to two pH units. There is little known about how this pH-change affects the light-harvesting complexes. In this study, we report multiscale simulations using a computationally efficient density functional tight-binding framework to investigate the spectroscopy and excitation energy transfer in the PC645 complex. Complementary experiments were conducted using both steady-state and time-resolved spectroscopic measurements at low, neutral, and high pH values. Our study shows that (de)­protonation of specific bilin pigments, namely, the mesobiliverdins (MBVs), modulates the excitation energies, excitonic couplings, and spectral densities. These changes cause excitation transfer rates to increase by up to a factor of two to three, leading to pH-dependent energy transfer pathways in the complex. Using this model, we calculated the pH-dependent fluorescence quantum yield of the system, obtaining quantitative agreement with the experimental results. These computational simulations, supported by experiments, identify MBVs as a more prominent excitation sink than previously realized, and that this role is tuned by pH.

## Graphical abstract

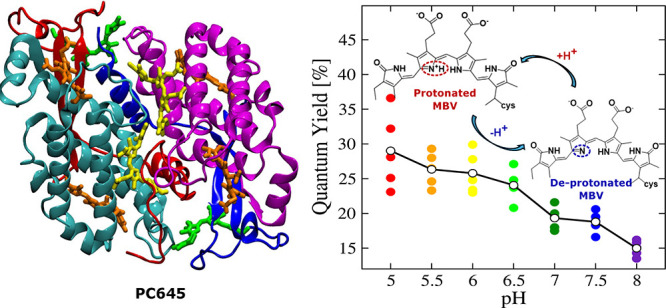

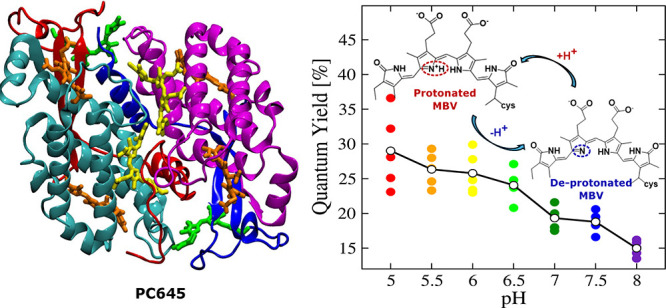

## 1 Introduction

Cryptophytes are unicellular algae found across diverse aquatic habitats, including marine, brackish, and freshwater environments. They are capable of harvesting sunlight under water and transferring this energy via linear, straight-chain tetrapyrrole chromophores known as bilins. These bilins are embedded within phycobiliproteins, which function as soluble light-harvesting antennae localized in the acidic thylakoid lumen and complement membrane-bound chlorophyll-*a*/*c* antenna complexes.^1–8^ The strong photosynthetic activity of these algae under very low-light conditions has attracted a lot of attention as researchers seek to reveal their light-harvesting mechanisms.^2,9–11^ The composition and content of bilins, and also the protein sequence, are responsible for the variation in the spectral properties of different phycobiliproteins.^5,12,13^ In particular, the insertion of a single aspartate residue on the α subunit of some phycobiliproteins results in a rotation by approximately 73°, leading to an almost 2-fold symmetry in the quaternary structure.^12^ Furthermore, the electronic interactions between the two central bilins vary considerably between the two structural conformations, referred to as the open and closed forms. These conformational differences critically influence the spectral properties of the system and govern its energy transfer dynamics, thereby playing a pivotal role in optimizing light-harvesting efficiency under varying environmental conditions.^12,14–17^

In this study, we have investigated the Phycocyanin 645 (PC645) complex, extracted from the cryptophyte algae *Chroomonas* sp. *CCMP270* (PDB code: 4LMS)^12^ with its structural details shown in Figure 1. This protein forms a homodimer consisting of two αβ subunits.^12^ Specifically, the (α_1_α_2_)­ββ structure comprises four chains: A, C (α_1_α_2_) and B, D (ββ) as described in the Protein Data Bank. In each α subunit, a mesobiliverdin (MBV) is covalently attached, while each β subunit binds one dihydrobiliverdin (DBV) and two phycocyanobilins (PCB) through cysteine residues.^18^ The MBV and PCB bilins are bound through a single cysteine linkage, while the DBV pigment is attached to the protein via two cysteine residues. As a result, the PC645 complex consists of two strongly coupled central bilins, i.e., DBV_50/61*B*_ and DBV_50/61*D*_, along with six peripheral bilins, including two MBV molecules (MBV_18*A*_ and MBV_18*C*_) and four PCB pigments (PCB_82*B*_, PCB_158*B*_, PCB_82*D*_, and PCB_158*D*_). These molecules are all linked to specific cysteine residues and associated with protein chains, creating the closed form of the PC645 complex (see Figure 1). One has to note that bilins can exist either in a protonated or in a deprotonated form, depending on the protonation states of the nitrogen atoms in the two central pyrrole rings and their coordination with nearby protein residues or water molecules (see Figures 1 and 2).

**Figure 1:**
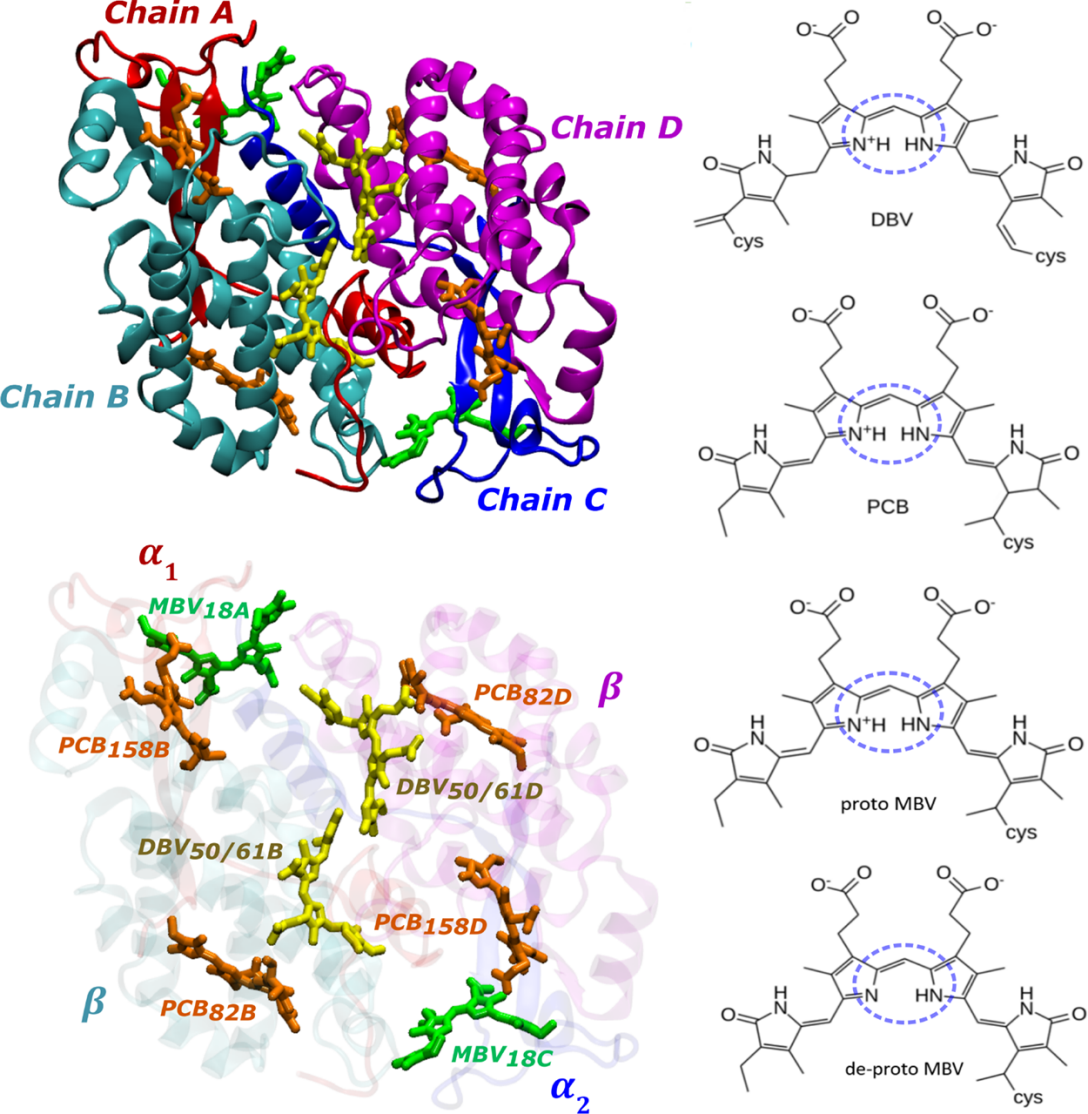
Left: Structure of the PC645 complex which contains eight bilin pigments (DBV in yellow, MBV in green, and PCB in orange) in two αβ subunits with chain A in red, chain C in blue, chain B in cyan, and chain D in magenta. Each α subunit binds an MBV, while each β subunit binds one DBV and two PCBs. Right: Chemical structures of the pigments presented in the PC645 complex. Both the DBV and PCB pigments are assumed to be protonated at their central pyrrole rings (indicated by blue dashed circles), while two possible protonation states of the MBV molecules are considered in the present study.

Previous studies by Scholes and co-workers have shown that the absorption spectrum of this complex exhibits two distinct peaks: a broad, lower-energy peak primarily associated with the PCB pigments and a higher-energy peak largely influenced by the strongly coupled central DBV molecules.^12^ This electronic coupling splits the DBV absorption bands at 585 nm into two exciton states labeled DBV+ and DBV-. Additionally, the MBVs are responsible for the 610 nm band observed in the middle of the spectrum, although its peaks are not prominent in the absorption profile. The PCBs, on the other hand, are responsible for the absorption near 645 nm. Previous studies reported that the fastest downhill energy transfer in PC645 occurs within approximately 600 fs, transferring energy from the higher-energy donor DBVs to the lower-energy terminal emitter PCB_82_.^9,19–22^ The rapid energy transfer is attributed to the interplay between an excitonic coupling and spectral overlap, which are inseparable on the relevant time scales, pointing to the role of vibronic coupling.^11,23^ Vibrations of bilin pigments have been recognized as crucial for excitation energy transfer in cryptophyte light-harvesting (LH) complexes,^11,22,24^ such as the transfer from DBV to PCB_82_. These vibrations enhance the spectral overlap integral by shaping the vibronic progressions in the donor’s emission spectrum and the acceptor’s absorption spectrum. In addition, the recent broadband pump–probe spectroscopy with chirped excitation pulse reveals that the energy transfer rate is influenced by initial state preparation.^25^ It is worth noting that most previous experiments were conducted under neutral conditions, with the pH ranging between 7 and 7.5.^9,22,25^ However, Corbella et al.^26^ performed pH-dependent absorption measurements on PC645 and other related cryptophyte phycobiliproteins, including PE545, PC577, and PC630. In their study, they measured linear spectra change over a broad pH range from pH 4.6 to 9.0. These observations did set an important precedent for the present work, which extends the pH-dependent steady-state spectra by directly examining how (de)-protonation of the MBV pigments modulates the corresponding excitation energies, spectral densities, energy-transfer rates, and excited state quenching.

Since the crystal structures were reported,^3,12^ numerous quantum chemical calculations have been conducted to investigate the exciton transfer mechanism and spectral properties of cryptophyte complexes.^17,23,26–29^ Moreover, several computational and experimental studies have predicted that the vibrational motion of the chromophores is correlated through electronic coupling, facilitating energy transfer within cryptophyte algae.^27,30–33^ To model such algae on an atomistic level, classical molecular dynamics (MD) simulations followed by excited-state calculations are often the basis of the theoretical calculations. These simulations provide essential parameters, such as the excitation energies (also known as site energies), excitonic couplings and the so-called spectral densities. A spectral density represents the coupling between the primary modes and the bath modes. In the present case, the former are the energy gaps between the ground and first excited states of the pigment molecules. The latter not only include the modes of the surrounding molecules but also the intramolecular vibrations. Accurately determining these intricate properties is crucial for studying phycobilin LH complexes, where vibrations play a pivotal role in facilitating the excitation energy transfer.^23^ Classical MD simulations followed by site energy calculations can introduce artifacts in spectral densities, particularly in the high-frequency region of intramolecular vibrations due to inaccuracies in the force fields and the so-called geometry-mismatch issue.^34^ To address this issue, alternative approaches, such as the vertical gradient approximation projecting onto the basis of normal modes^28^ and DFT-based QM/MM MD simulations^23^ have been considered to determine the electronic-vibrational contributions as well as the reorganization energies to study the energy transfer process in the PC645 complex. However, such approaches are computationally demanding for large molecules like billins. In this context, our recently developed multiscale technique, utilizing the numerically efficient Density Functional based Tight-Binding (DFTB) approach,^35^ demonstrates an excellent compromise between accuracy and numerical efficiency in spectral density calculations for plants,^36–38^ bacteria^39^ and diatom algae.^40^ This method involves performing QM/MM MD simulations at the SCC-DFTB3 level of theory^41^ using the 3OB-f parameter set,^42^ followed by excited-state calculations based on time-dependent long-range corrected DFTB (TD-LC-DFTB) with the OB2 parameter set^43^ in a QM/MM framework to obtain the spectral density. We have applied this protocol in the present study for the PC645 complex to extract the site energies, dipole moments and spectral densities of all bilin molecules.

**Figure 2:**
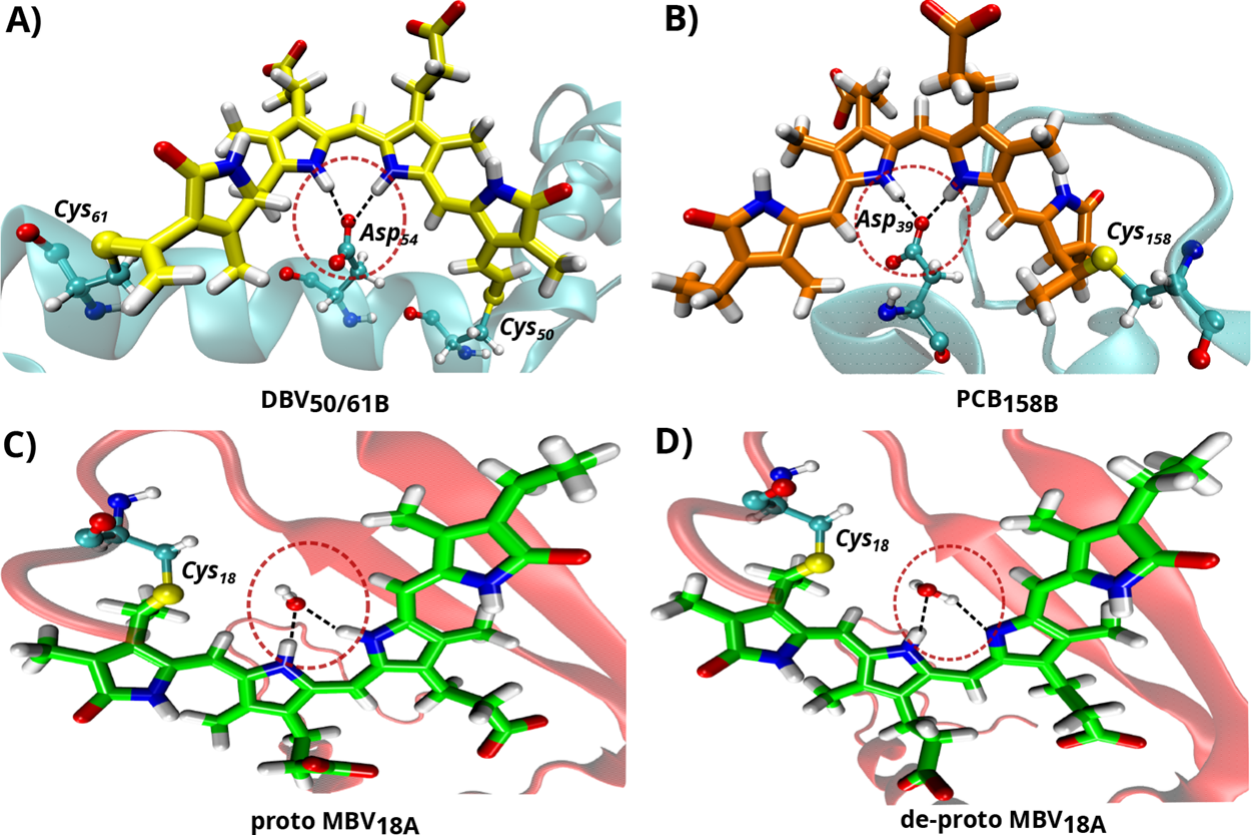
Examples of protonated and deprotonated bilin molecules interacting with their local protein environments: (A, B) Protonated DBV_50/61*B*_ and PCB_158*B*_ pigments coordinate with aspartate residues (Asp 54 and Asp 39) while (C) protonated and (D) deprotonated MBV_18*A*_ molecules coordinate with a water molecule (see the dashed red circle).

In contrast to the situation with red algae, cryptophyte phycobiliproteins are not organized into phycobilisomes, but are located in the thylakoid lumen,^44^ where the pH value exhibits a substantial light-dependent variation, ranging from approximately 5.5 to 7.5.^45,46^ Under such varying pH conditions, the structure of the bilin molecules permits the protonation and deprotonation of the nitrogen atom in the central pyrrole ring (see Figure 1), a factor that significantly influences the excited-state properties and subsequent energy transfer processes.^28^ Consequently, we have thoroughly examined the precise positioning and orientation of each bilin within the corresponding protein binding pocket. The available crystal structure reveals that the central pyrrole of all DBVs and PCBs interacts with an aspartate residue in the protein, suggesting that these bilins are fully protonated due to their ability to form hydrogen bonds.^12,47^ In contrast, the MBVs in PC645 are coordinated with water molecules but lack a definitive orientation, leaving the protonation state of the MBVs uncertain.^48^ Figure 2 illustrates examples of the protonated and deprotonated forms of DBV_50/61_, PCB_158_, and MBV_18*A*_, highlighting their interactions with the local protein environment and their respective cysteine linkages in the chains A and B. The other bilin molecules show similar interactions. As a pH-dependent behavior of protein complexes in the lumen might be connected to nonphotochemical quenching (NPQ), a detailed analysis of the different protonation states of the PC645 complex and of the excitation energy transfer with the complex might yield evidence in that direction as well. At the same time, the only report of experiments on NPQ in cryptophytes indicates that NPQ in cryptophytes occurs in the chlorophyll-*a*/*c* antennae instead of the phycobiliproteins.^45^

Absorption spectra calculations by Coker and co-workers^28^ suggest that the MBVs in PC645 are deprotonated, achieving a better agreement with experimental data, while quantum chemical calculations and pH-dependent absorption spectroscopy studies by Curutchet and co-workers^26^ support a preference for the fully protonated state of the bilins in cryptophyte complexes under physiological conditions. However, it is important to note that none of the absorption line shapes based on atomistic simulations fully reproduce the experimental absorption spectra rather they reproduce two distinct peaks corresponding to the DBV and PCB bilins. Thus, to resolve this ambiguity of the MBV protonation states, we considered both the protonated and deprotonated states of the MBVs in PC645 in this study, while keeping all other bilin molecules remain fully protonated. Using a multiscale approach as previously described, we calculated site energies, excitonic couplings, and spectral densities, which were subsequently used as key inputs to determine the Förster energy-transfer rates between specific pigment pairs. Moreover, both steady-state and time-resolved spectroscopic experiments, specifically linear and transient absorptions, fluorescence, and Time-Correlated Single Photon Counting (TCSPC), were carried out to determine absorption spectra, real-time dynamics, quantum yields and excited state lifetimes under various pH conditions (pH 5.0–8.0). The observed pH-dependent changes in the linear and transient absorption spectra are in good agreement with the calculated excitonic energies and transition dipole moment, supporting a protonation–deprotonation event of the MBV bilin. In addition, the measured quantum yields show strong consistency with the theoretical analysis, indicating a quenched form of the PC645 complex at high pH. Experimentally, quenching of around 40–50% is observed, which has been reproduced by the theoretical modeling as well.

Both theoretical predictions and experimental measurements indicate that MBVs can act as pH-responsive regulatory elements. The protonation state of the MBVs strongly influences the excitation energy landscape of the PC645 complex, as well as the spectral density, leading to a 2–3 fold variation in excitation transfer rates between DBVs and MBVs. The relaxed E_0–0_ energies suggest that MBVs function as terminal excitation sinks on longer time scales at both pH conditions, whereas PCB_82_ might act as the terminal emitter only at neutral pH, as inferred from the vertical excitation energies on ultrafast time scales. This finding contrasts with previous reports that assigned PCB_82_ as the sole terminal emitter within the PC645 complex. The observed quenching mechanism is particularly intriguing, as it parallels mechanisms identified in membrane-bound antenna systems of higher plants, where excitonic coupling between pigment pairs plays a central role in energy dissipation at low pH.^49^ In contrast, in the water-soluble PC645 complex, pH-dependent quenching appears to arise from modulation of vibrational degrees of freedom of the bilin chromophores, which in turn influence site energies and excitonic couplings predominantly at high pH. Moreover, our structural analysis of available structures of closed-form phycobiliproteins reveals a conserved architectural motif in which a specific bilin chromophore can undergo protonation–deprotonation, suggesting that high-pH-induced quenching may represent a general mechanism in these systems.

## 2 Results and Discussion

### 2.1 Effect of Different Protonation States on the Hamiltonian

In a first step, we describe the computational study of the PC645 complex. As a basis of such an analysis of a pigment–protein complex in the framework of open quantum systems, one usually needs to construct the excitonic Frenkel Hamiltonian^50^ as

$$H=\sum_{m}E_{m}|m\rangle \;\langle m|+\sum_{m\neq n}V_{mn}|n\rangle \;\langle m|$$ 1

where the diagonal elements *E*_*m*_ denote the site energies of pigments *m* and the off-diagonal elements *V*_*mn*_ the respective interpigment couplings. This Hamiltonian is defined within the single-exciton manifold, meaning it does not account for de-excitations to the electronic ground state or for double (and higher-order) excitations. The site energies represent the vertical excitation energies to the lowest, i.e., the S_1_, excitation energies of the pigment molecules along the trajectories. In this context, these energies are referred to as Q_*y*_ excitation energies. While the Q_*y*_ state in Chl and BChl pigments is well-defined along the trajectory and corresponds exclusively to the S_1_ state in TD-DFTB calculations, bilin pigments can exhibit a slight state mixing along the trajectory. To address this, we extracted the Q_*y*_ excitation energies along the trajectory by analyzing, in addition, the associated transition dipole moment.

After preparing the system (see Computational Protocol for details), we first calculated the excitation energies for the protonated and deprotonated PC645 complexes using the TD-LC-DFTB method along 1 ns DFTB/MM MD trajectories within a QM/MM framework. The energy landscape based on the average values is shown in Figure 3, while the associated distributions are shown in Figure S2 in the SI. Additionally, we compared our results with those reported by Ozaydin et al.,^29^ Lee et al.,^28^ and Mirkovic et al.^19^ for the same system. While TD-LC-DFTB has shown promising performance for studying BChl and Chl pigments in light-harvesting complexes of bacteria^51^ and plants,^36–38^ it has not yet been applied to bilin pigments. Thus, to benchmark the findings of this study, we additionally calculated site energies within the TD-LC-DFT framework (CAM-B3LYP/Def2-TZVP) for QM/MM-optimized geometries. These results were compared to the TD-LC-DFTB calculations performed on QM/MM optimized structures, as well as to the average site energies calculated over a 1 ns QM/MM MD trajectory, as illustrated in Figure S1. We found that the two methods based on the DFT and DFTB approaches exhibit a consistent trend with only minor quantitative differences. Nevertheless, this agreement underscores the reliability of the computationally efficient TD-LC-DFTB method for studying bilin molecules.

**Figure 3:**
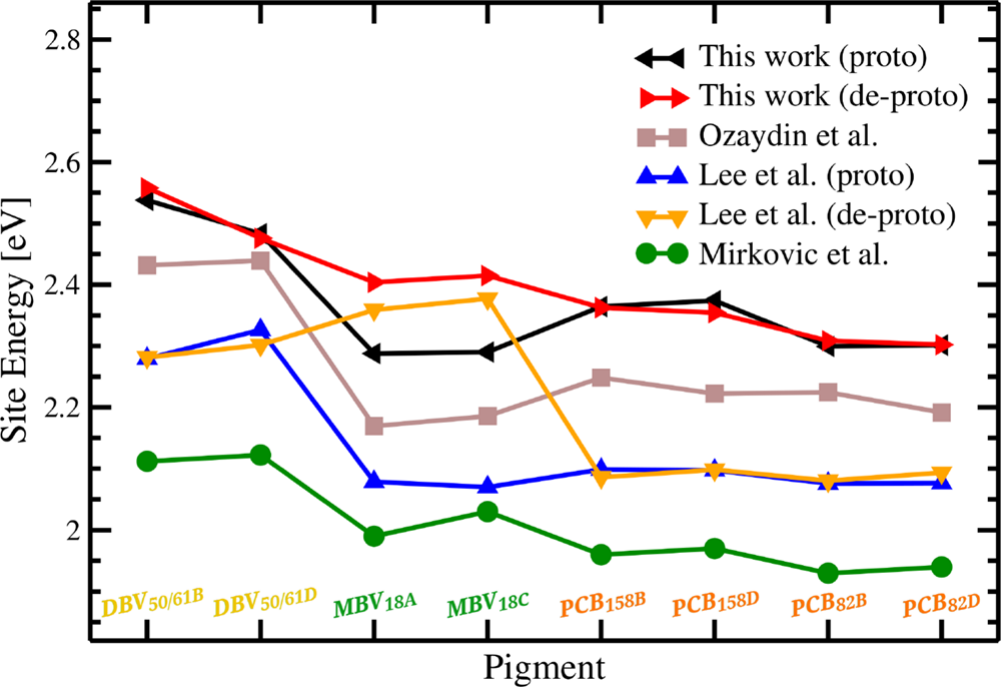
Average site energies of the pigments in the protonated and deprotonated forms of the PC645 complex calculated using TD-LC-DFTB along a 1 ns QM/MM MD trajectories and compared to previous results from Ozaydin et al.,^29^ Lee et al.,^28^ and Mirkovic et al.^19^

The energy ladder shows that the DBVs, which are two strongly interacting chromophores in the center of the system, have the highest site energies and may act as energy starting points in the system. Compared to the DBVs, the MBV and PCB molecules have lower excitation energy gaps. Among them, PCB_82_ has the lowest site energy when the MBVs are deprotonated, making this pigment the terminal emitter. However, when the MBVs are protonated, their site energies drop significantly, making them the lowest in the energy profile. This matches findings by Coker and co-workers,^28^ who observed a similar trend when comparing protonated and deprotonated states.

The excitation energies calculated by Mirkovic et al. in their earlier study show a trend similar to that one obtained for the deprotonated systems investigated in the present work.^19^ In their study, the excitation energies were computed using configuration interaction singles (CIS) calculations based on crystal structures, while environmental effects were treated implicitly using the polarizable continuum model (PCM). Based on these results, several subsequent experimental studies assigned the intermediate-energy absorption bands to the MBVs.^9,12^ However, it should be noted that all bilins were deprotonated in the work by Mirkovic and co-workers with only one of the two central pyrrole nitrogen atoms being protonated. In contrast, the present study specifically investigates MBV pigments in both protonated and deprotonated forms, while all remaining bilins are kept fully protonated, following a strategy similar to that employed by Lee et al.^28^ Subsequently, combined quantum chemical calculations and experimental absorption spectroscopy by Curutchet and co-workers^26^ supported a preference for fully protonated bilins in cryptophyte antenna complexes under physiological conditions. More recently, they employed QM/MMPol calculations along DFT-based QM/MM molecular dynamics trajectories and demonstrated that the MBV pigments possess the lowest site energies in the PC645 complex when all bilins are fully protonated. Furthermore, the present results, together with previous studies by Lee et al.^28^ and Kosenkov et al.,^33^ consistently support the same energy ladder, despite differences in the computational details. Such protonation-dependent changes in the energy landscape significantly affect both the composition of excitonic states and the resulting absorption bands. These effects will be discussed in more detail in the following section.

As a second component of the Hamiltonian and as shown in eq 1, the excitonic coupling values between all pigment pairs were computed based on the so-called TrESP approach (Transition Charge from Electrostatic Potential).^52^ The results are detailed in Section S4 in the Supporting Information. However, as can be seen, the excitonic couplings are less significantly affected by the pH value, since the transition dipole moment is only slightly altered by the protonation and deprotonation events, as shown in Table S5 in the Supporting Information.

Overall, the protonation induces a pronounced shift in the excitation energy of the MBV bilins in the PC645 complex, resulting in a significantly different energy profile among the pigments. Consequently, the MBV pigment may act as a regulator, providing alternative possibilities for exciton transfer pathways within the PC645 complex based on its protonation state. Despite a considerable variation in excitation energy, the coupling analysis reveals only a minor change, likely since the MBV bilins only show minimal changes in their transition dipole moments when changing their protonation states. The transition dipole moments for all pigments are provided in Table S5 in the Supporting Information.

### 2.2 Excitonic States at Different Protonation States

After constructing the excitonic Hamiltonian, we determined the excitonic states and the contributions of the individual pigments to these states based on the excitonic Hamiltonians given in Tables S1 and S2 for the protonated and deprotonated cases. Table 1 presents the excitonic energies, while Tables S3 and S4 in the SI list the eigenvectors, which describe the contribution of each pigment to the respective excitonic states. These tables reveal that in both the protonated and deprotonated systems, the DBV pigments contribute most significantly to the highest-energy excitonic state. PCB_82_ contributes the most to the lowest excitonic state in the deprotonated form, whereas in the protonated form, MBV bilins dominate the lowest-energy excitonic state. This suggests that the protonation of MBV significantly alters the energy distribution of the exciton states, at least on an ultrafast time-scale. However, the ultimate direction and efficiency of energy transfer within the complex are dictated not only by these static excitonic contributions, but also by the system-bath interactions.^53^ To quantify the differences in the associated excitation energy transfer rates, a detailed rate calculation is necessary to gain a mechanistic understanding of the energy transfer within the PC645 pigment network. Therefore, after determining the spectral densities, the Förster transfer rates have been calculated and are reported below, while particular attention has been given to the comparison between the DBV/MBV and DBV/PCB_82_ pairs, given that their competition plays a significant role in the energy transfer process.

**Table 1:** Excitonic Energies and Pigment Contributions for the Protonated and Deprotonated States of the PC645 Complex^a^

	energy (cm^–1^)		pigment contribution	
exciton state	**proto**	**de-proto**	**proto**	**de-proto**
1	18381	18556	MBV_18*A*_ (53%), MBV_18*C*_ (22%), PCB_82*B*_ (10%), PCB_82*D*_ (12%)	PCB_82*D*_ (98%)
2	18420	18611	MBV_18*A*_ (28%), MBV_18*C*_ (53%), PCB_82*B*_ (12%)	PCB_82*B*_ (98%)
3	18575	18964	MBV_18*C*_ (14%), PCB_82*B*_ (59%), PCB_82*D*_ (24%)	PCB_158*D*_ (97%)
4	19091	19025	PCB_158*B*_ (94%)	PCB_158*B*_ (90%)
5	19170	19415	PCB_158*D*_ (94%)	MBV_18*A*_ (88%)
6	18595	19501	MBV_18*A*_ (12%), PCB_82*B*_ (19%), PCB_82*D*_ (59%)	MBV_18*C*_ (92%)
7	19736	19767	DBV50/61*B* (29%), DBV50/61*D* (71%)	DBV50/61*B* (19%), DBV50/61*D* (76%)
8	20779	20858	DBV50/61*B* (71%), DBV50/61*D* (29%)	DBV50/61*B* (79%), DBV50/61*D* (20%)

a The excitonic states are listed from lowest to highest energy. Only pigment contributions above 10% are included.

### 2.3 Spectral Densities of the Individual Pigments

Spectral densities describe how environmental modes influence the dynamics of quantum systems and can be derived from site energy fluctuations within the framework of the open quantum system theory.^50^ Several theoretical and experimental methods exist to determine this quantity. In the present calculation, we extract it using a cosine transform of the autocorrelation of the site energy fluctuations^54^

$$J_{m}(\omega )=\frac{\beta \omega }{\pi }\int_{0}^{\infty }dtC_{m}(t)\;\cos(\omega t)$$ 2

where β = 1/(*k*_B_ *T*) denotes the inverse temperature and *C*_*m*_(*t*) the site-energy autocorrelation function. This function can be computed from the time series of energy gaps as

$$C_{m}\left(t_{l}\right)=\frac{1}{N-l}\overset{N-l}{\underset{k=1}{\sum }}\Delta E_{m}\left(t_{l}+t_{k}\right)\Delta E_{m}\left(t_{k}\right)$$ 3

where *E*_*m*_ denotes the site energy of pigment *m* and Δ*E*_*m*_ = *E*_*m*_ – ⟨*E*_*m*_⟩ is the deviation of the site energy from its average value ⟨*E*_*m*_⟩. Here, *N* is the total number of snapshots or the length of the time window, and *l* is the autocorrelation time lag.

The spectral densities of the individual pigments in PC645 were determined from three sets of QM/MM MD trajectories, and their averages are shown in Figure 4. The average over all pigments, together with the spectral density calculated by Blau et al. from ref 23, is presented in Figure S4 of the SI for both the protonated and deprotonated states. In both cases, the DBV and PCB pigments remain protonated, while the MBV molecules can exist in either a protonated or deprotonated state. This variation depends on the interaction between the nitrogen atoms in the central pyrrole ring and the aspartate residue in the local protein environment, as discussed above. Consequently, the spectral densities of the DBV and PCB molecules are similar in both the protonated and the deprotonated version of PC645, with minor differences attributed to sampling issues. When comparing the spectral densities for the protonated and deprotonated MBV pigments, significant differences emerge in the high-frequency region, mainly around 1200–1700 cm^–1^. This region is primarily influenced by intramolecular vibrations arising from modes involving C=C, C=N, and C=O bond vibrations. The deprotonated MBV molecules exhibit more distinct vibrational peaks compared to the more rigid protonated form. This is likely because the protonated MBV molecule preserves the aromaticity of the central pyrrole rings, with highly delocalized electrons that render the structure more rigid. In contrast, deprotonation disrupts this aromaticity, leading to a more flexible molecular structure. These changes restrict certain vibrational modes, thereby reducing the amplitudes of the vibrational peaks. Moreover, the structural changes also lead to variations in the reorganization energies, as discussed in Section S7 in the Supporting Information.

**Figure 4:**
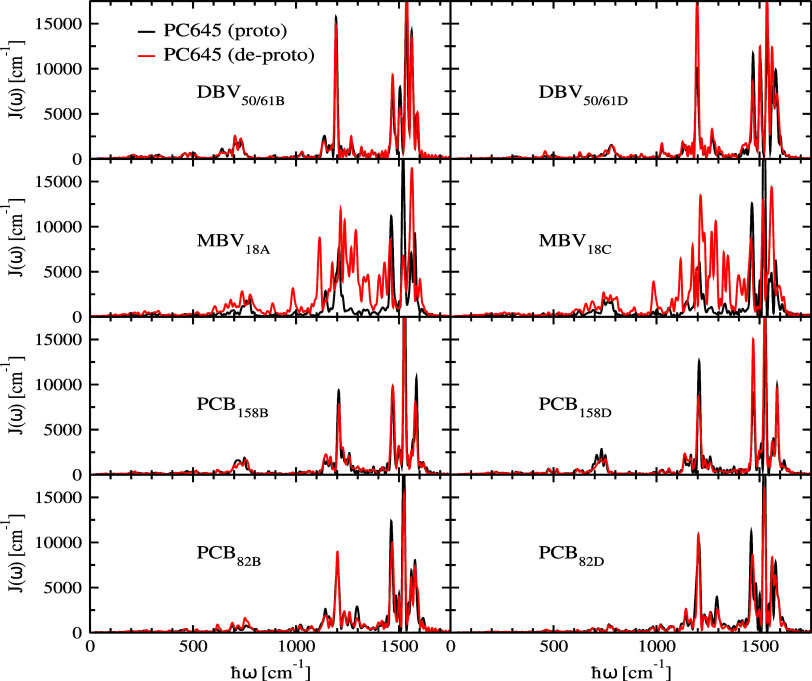
Spectral densities of the individual pigments based on two different sets of simulations for the PC645 complex: once with the MBV molecules protonated (black) and once deprotonated (red).

In the following sections, the experimentally measured spectroscopic properties and fluorescence quantum yields are reported. The theoretical calculations presented above indicate that distinct protonation states of MBV within the PC645 complex give rise to substantial changes in both its intramolecular vibrational modes and the overall excitonic energy landscape. Although these microscopic quantities are not directly accessible experimentally, their impact on observable properties, such as absorption spectra and excited state lifetimes, provides a direct link between theory and experiment.

### 2.4 pH-Dependent Steady-State Absorption

To experimentally determine whether the pH affects the steady state properties of the phycobiliprotein, we first measured the absorption spectra of the system at low and high pH. Figure 5 shows the normalized absorbance spectra of the PC645 complex between pH 6.0 and 8.0. The pH levels were selected based on theoretical predictions for the protonation state of the MBV bilins.^26^ At pH 6.0, the MBVs are expected to be protonated, while at pH 8.0, they are expected to be deprotonated. Increasing pH systematically alters the relative intensities and lineshapes of the bilin absorption features showing minor changes below pH 6.5 and more pronounced variations above this value, in qualitative agreement with previous experimental measurements.^26^ In particular, the low-energy band at around 650 nm decreases significantly in relative intensity as the pH increases. The midenergy region from roughly 610 to 640 nm also shows a reduced oscillator strength and an increased spectral broadening at higher pH.

**Figure 5:**
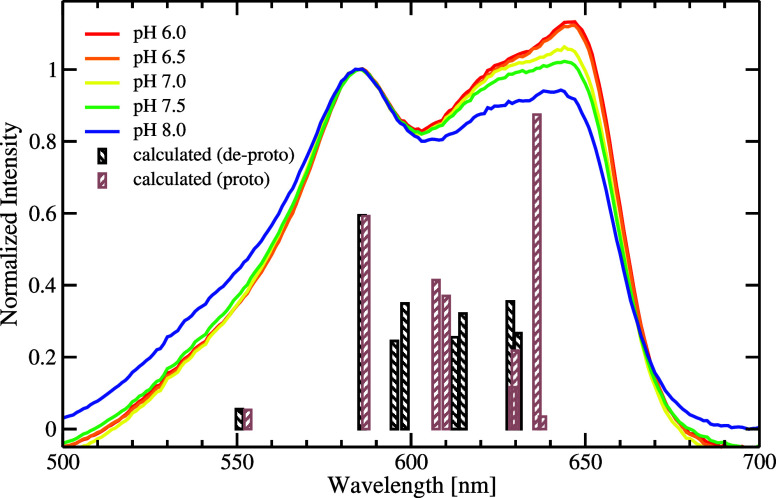
Normalized absorbance spectra of PC645 at five different pH levels, and the calculated excitonic stick spectra. The experimental curves have been normalized with respect to the peak at 585 nm. The stick spectra are shifted by 2700 cm^–1^ to roughly match the experimental one.

To facilitate comparison with the calculated excitonic properties, we present the excitonic energies as stick spectra in Figure 5, with line heights proportional to the squared transition dipole moments evaluated in the excitonic basis rather than the site basis. The two high-energy excitonic states at short-wavelength correspond to the experimental absorption peak at approximately 585 nm and are dominated entirely by DBV contributions (see Table 1). Their absorption intensities are essentially identical in the two variants of the system. In contrast, large differences can be observed for the two low-energy excitonic states at long wavelengths, and our theoretical analysis suggests that these differences originate from the protonation-induced rearrangement of the excitonic energy landscape. As summarized in Table 1, in the deprotonated system states 1 and 2 are predominantly attributable to PCB_82*D*_ and PCB_82*B*_ with a contribution of 98%. When the pH is lowered, causing MBV within the complex to become protonated, both the PCB_82_ and the MBV molecules contribute to these two excitonic states, i.e., the exciton delocalization increases. However, MBV provides the dominant contribution in this pairwise delocalization, i.e., exceeding a contribution of 75%. In addition, MBV exhibits an increased transition dipole moment, as reported in Table S5 in the Supporting Information, thus resulting in a marked enhancement of the overall absorption intensity and width.

### 2.5 pH-Dependent Femtosecond Transient Absorption

To directly probe the initial excited-state population dynamics and energy transfer pathways within PC645, we performed femtosecond transient absorption (fs-TA) spectroscopy under varying pH conditions.

The spectra are characterized by a ground-state bleach (GSB) spanning from 560 to 670 nm with multiple features and an excited-state absorption (ESA) from 670 to 720 nm (see Figure 6). Across all pH conditions, we observed a prominent negative peak centered around 585 nm at early times. It subsequently decays as the low-energy peak grows, particularly at ∼650 nm. This temporal evolution shows that the high-energy exciton manifold gets populated immediately following excitation and then relaxes into the low-energy states, which is consistent with the results from Table 1 where the high-energy excitons (excitonic states 7 and 8) are assigned to the DBV bilins, while the low-energy excitons (excitonic states 1 and 2) correspond to the energy acceptors. Table 1 further indicates that the composition of the low-energy states depends on the protonation conditions. In an acidic environment, there is a substantial MBV contribution and a relatively smaller PCB_82_ contribution. In a basic environment, PCB_82_ contributes to the low-energy peak primarily. However, these changes in pigment contribution are not clearly resolved in the TA spectra, e.g., as peak shifts or large changes in relative intensities between peaks. Instead, the spectra at longer time delays exhibit similar lineshapes across various pH levels. This suggests that the protonation-dependent effect is most likely reflected through differences in the population dynamics, which can arise from variations in the energy transfer kinetics and system-environment interaction.

**Figure 6:**
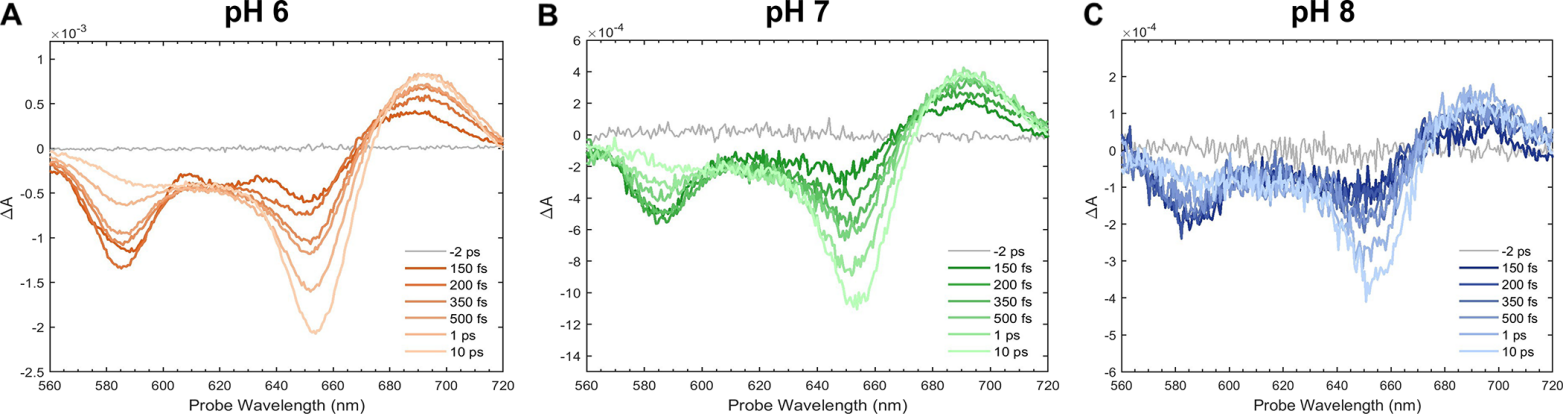
TA spectra of PC645 in 50 mM phosphate buffer at (a) pH 6.0, (b) pH 7.0, and (c) pH 8.0, when excited at 560 nm and probed in the visible region.

Due to the spectral overlap of signals from multiple bilins and limited time resolution, it is very challenging to extract distinct lifetimes for individual pigments. Only a subtle distinction at pH 6.0 is observed in the TA spectra, where an additional transient feature near ∼620 nm appears at an early time delay. This feature is significantly weaker or absent at pH 7.0 and 8.0. While this small difference suggests changes in population dynamics in an acidic environment, its specific assignment is not conclusive. Overall, our TA results qualitatively infer that the bilin protonation pattern may influence energy transfer pathways in PC645.

### 2.6 pH-Dependent Fluorescence Quantum Yields and Rate Analysis

The agreement between experimental linear and transient absorption and calculated excitonic energies clearly suggests that protonation and deprotonation events occur in the PC645 complex. In the next step, we analyze the impact of this mechanism on the photophysical properties of the system. To this end, we perform fluorescence experiments and absolute quantum yield measurements to quantify changes in the emission efficiency at different pH levels, as presented in Figure 7 and in Table S7 in the Supporting Information. As one can see in Figure 7, the quantum yield monotonically decreases with increasing pH. At pH 8.0, where the MBVs are predicted to be deprotonated, the average quantum yield is calculated to be 15%, representing a roughly 48% decrease relative to pH 5.0. This reduction in quantum yield reflects intrinsic changes in the photophysical properties of the bilin network because the protein integrity is maintained across the different pH values (see section S8 in the Supporting Information). Thus, we extend our theoretical model to calculate rate constants, followed by quantum yield calculations in the next section, in order to test how the protonation–deprotonation event of MBV connects with the experimental quantum yield. However, it should be noted that the quantum yield in the PC645 complex is relatively low compared to antenna complexes in higher plants,^55^ suggesting that various nonradiative processes might already occur within the system (e.g., internal conversion, photoisomerization, etc.) as has been reported for the PE545 complex.^56^

**Figure 7:**
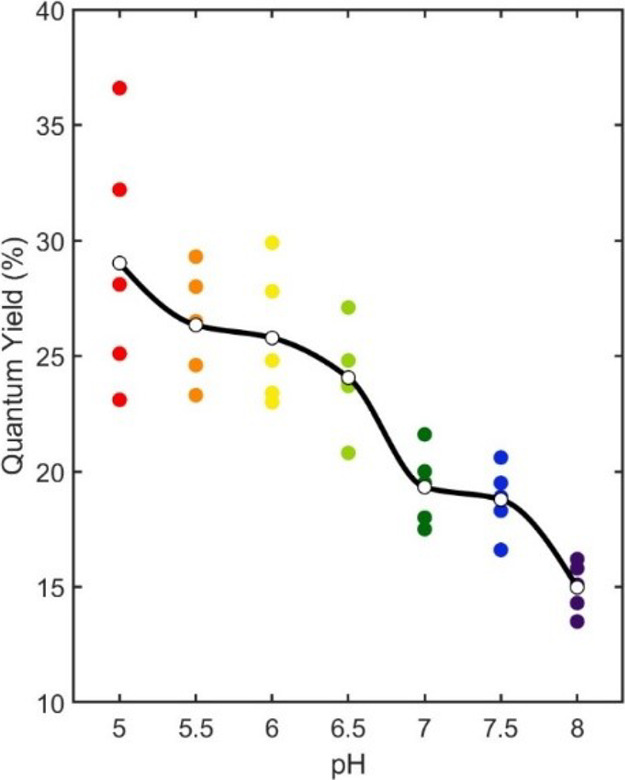
Quantum yields of PC645 at seven different pH values. The solid dots show the raw data points while the hollow ones depict the average quantum yields from five replicating measurements at each pH level.

To further probe the excited-state event of the system, we performed TCSPC measurements to determine the fluorescence lifetimes of the complex at different pH values. These measurements also allow us to assess whether changes in the fluorescence lifetime accompany the observed decrease in quantum yield. Representative decay profiles along with their corresponding fits are shown in Figure 8, and the extracted fitting parameters are summarized in Table 2. The TCSPC decay traces were analyzed using single exponential fits at pH 6 and 7, and for pH 8, a biexponential fit of the form

$$I(t)=\sum_{i}A_{i}e^{-t/\tau_{i}}$$ 4

where τ_*i*_ denotes the effective fluorescence lifetimes and *A*_*i*_ are their corresponding amplitudes. In the case of pH 8, the shorter lifetime component of roughly 1.39 ns accounts for about 95% of the emission intensity and is therefore taken as the primary emission lifetime of the whole complex for comparison at varying pH levels. Across pH 6.0 to 8.0, the dominant lifetime decreases by only about 4%, from 1.45 to 1.39 ns, despite the much larger decrease in quantum yield.

**Figure 8:**
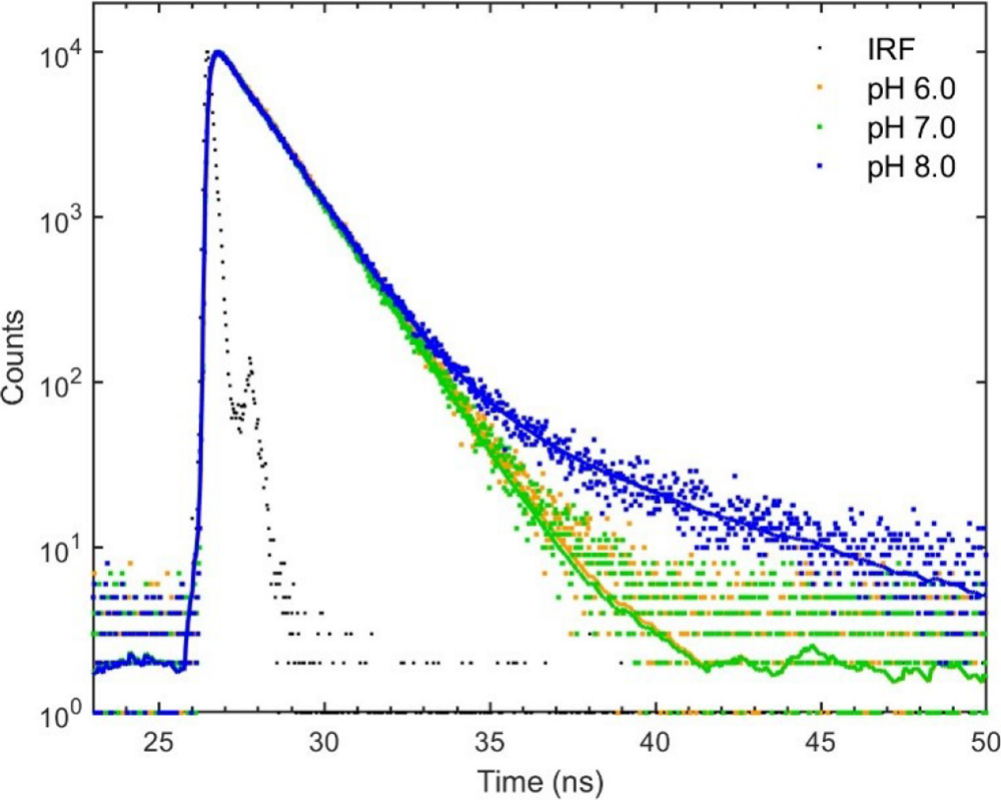
Time-correlated single photon counting results collected until a maximum of 10,000 counts was attained. The raw data points of PC645 at pH levels of 6.0, 7.0, and 8.0 are shown as dots with kinetic fits overlapping them as solid lines.

**Table 2:** Time Constants τ_1_ and τ_2_ and Intensity Weighting Factors *A*_1_ and *A*_2_ from a Kinetic Analysis of the Decays of PC645 at pH Levels of 6.0, 7.0, and 8.0 Monitored by TCSPC^a^

pH	τ_1_ (ns)	τ_2_ (ns)	*A*_1_	*A*_2_	χ^2^
6.0	1.45		0.127		1.16
7.0	1.42		0.127		1.18
8.0	1.39	5.95	0.126	0.00173	1.14

a The quality of each kinetic fit is indicated by the value of χ^2^.

Using the TCSPC measurements together with the fluorescence quantum yield values, we can extract both the radiative and nonradiative rates of the PC645 complex. Table 3 presents the photophysical parameters of PC645 at three different pH conditions, listing the quantum yields, lifetimes, and the corresponding decay rates. From these data, we find that the radiative rate decreases by 39% and the nonradiative rate increases by 20%, whereas the lifetime changes only modestly by about 4% when the pH is raised from 6.0 to 8.0. It should be noted that the measured lifetime reflects the dynamics of the entire complex rather than any single bilin, although it may be strongly influenced by an individual emitter or energy sink.

**Table 3:** Quantum Yields, Photoluminescence Decay Lifetimes, Total Decay Rates, Radiative Rates *k*_*r*_, and Nonradiative Rates *k*_*nr*_ of the PC645 Complex at Three Different pH Values

pH	average quantum yield (%)	τ (ns)	*k*_tot_ (s^–1^)	*k*_*r*_ (s^–1^)	*k*_*nr*_ (s^–1^)
6.0	25.8	1.45	6.90 × 10^8^	1.78 × 10^8^	5.12 × 10^8^
7.0	19.3	1.42	7.04 × 10^8^	1.36 × 10^8^	5.68 × 10^8^
8.0	15.0	1.39	7.19 × 10^8^	1.08 × 10^8^	6.13 × 10^8^

### 2.7 Excitation Transfer Rate and Quenching Mechanism

After detecting a sharp decrease in quantum yield of about 50% at high pH in fluorescence measurements, we constructed a rate model to reproduce this behavior from an atomistic perspective. To this end, we first take a look at the transfer rates between the pigments within the Förster resonance energy transfer (FRET) theory. We have chosen this method because the reorganization energies of the bilin molecules are much larger than the interpigment couplings, as we saw in earlier sections, i.e., λ_*m*_ ≫ *V*_*mn*_, placing the system in the incoherent hopping regime. In this regime, vibrational relaxation dominates over interpigment energy transfer, making Förster theory an appropriate description. In this approach, the rate of energy transfer between two pigments can be determined using the transfer rate constant *k*_*m*→*n*_ within the weak interpigment coupling limit as follows^57–60^

km→n=2ℏ2|Vmn|2Re∫0∞Fm*(t)An(t)dt 5

Here, *V*_*mn*_ denotes the excitonic coupling between pigments *m* and *n*. The donor fluorescence response function *F*_*m*_(*t*) and the acceptor absorption response function *A*_*n*_(*t*) are given in the time domain by^57,58^

$$F_{m}\left(t\right)=\exp\left[-i\left(\frac{E_{m}-2\lambda_{m}}{\hbar }\right)t-g_{m}^{*}\left(t\right)\right]$$ 6

$$A_{n}\left(t\right)=\exp\left[-i\left(\frac{E_{n}}{\hbar }\right)t-g_{n}\left(t\right)\right]$$ 7

In these expressions, *E*_*m*_ and *E*_*n*_ are the site energies of the donor *m* and acceptor *n*, respectively, and λ_*m*_ is the reorganization energy of the former. The line-shape function *g*_*m*/*n*_(*t*) can be computed from the spectral density *J*(ω) using^57,58^

$$g_{m/n}(t)=\int_{0}^{\infty }\frac{d\omega }{\hbar \omega^{2}}J_{m/n}(\omega )\left[\left(1-\cos(\omega t))\coth\left(\frac{\hbar \omega }{2k_{B}T}\right)+i(\sin(\omega t)-\omega t\right)\right]$$ 8

These expressions demonstrate that site energies, excitonic coupling values, and spectral densities are the key parameters required to calculate the excitation transfer rate. These quantities are provided as input parameters and are computed based on molecular simulations, as described in the previous sections.

In Figure 9, we present the calculated excitation transfer rates between the pigment pairs, depicted schematically for both the protonated (red) and the deprotonated (blue) complex. Here, we split the system shown in Figure 1 into two symmetrical clusters. One cluster comprises of the four bilins, DBV_50/61*D*_, MBV_18*A*_, PCB_158*B*_, and PCB_82*D*_, while the other cluster consists of the remaining four bilins. Among all chromophore pairs between these two clusters, only the DBV/DBV pair has a strong electronic coupling, whereas the coupling values between all other pairs are weak and contribute to energy transfer only in a negligible manner. Consequently, we restrict our analysis to the intracluster transfer processes and simplify the model to a four-site system. Furthermore, we have also calculated the energy transfer rates in both forward and reverse directions between pigment pairs using the specified input parameters. The main values are shown in black and green in Figure 9, and the complete rate matrices are provided in the Tables S8 and S9 in SI for the protonated and deprotonated cases. It is crucial to emphasize that the FRET represents a fundamentally incoherent methodology for analyzing multichromophoric systems.^61^ Unlike FRET, advanced computational techniques incorporating quantum coherence can substantially improve the calculation of excitation transfer rates, especially when one is concerned with the ultrafast dynamics.^62^ However, this study concentrates on qualitatively comparing relative rates within the pigment network. In this context, despite its possible limitations in quantitative accuracy, FRET remains an appropriate and effective method. Furthermore, we verified that this FRET model is consistent with detailed balance at thermal equilibrium (see Section S12 of the Supporting Information), supporting the robustness of the model.

**Figure 9:**
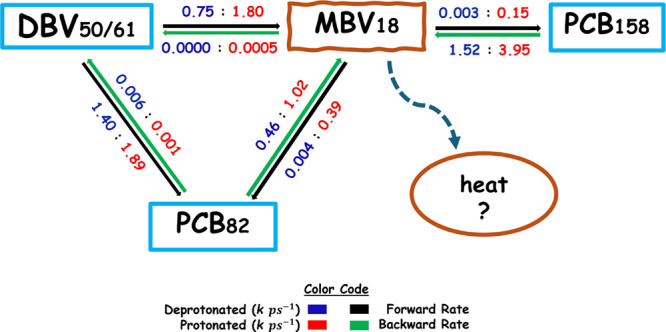
Schematic rate diagram of the pigment network showing forward rates in **black** and backward rates in **green** for different protonation states of the PC645 complex. Rates associated with the deprotonated complex are shown in **blue** while those altered in the protonated complex are highlighted in **red**.

Based on the calculated site energy profile as well as the excitonic energy analysis, the DBV pigments can be seen as the starting point of the excitation energy transfer. From there, the excitation energy is transferred to either the PCB_82_ or MBV_18_ pigments, depending upon the protonation event. The present results indicate that the protonation state of the MBV pigments strongly affects the preferred energy-transfer pathways. When the MBVs are deprotonated, the transfer rate within the DBV-PCB_82_ pair is significantly higher than that of the other pigment pairs, suggesting that on short time scales, the excitation energy preferentially transfers directly to PCB_82_ without passing through MBV. In contrast, in the protonated state, the transfer rate between DBV and MBV increases by a factor of two to three compared to the deprotonated case and becomes comparable to that between the DBV and PCB_82_ pigments. Consequently, two competing transfer pathways coexist: from DBV to MBV or to PCB_82_. These results are consistent with the site energy landscapes for the complex in its two protonation states, as shown in Figure 3; however, they apply primarily to the short-time dynamics. Furthermore, this is consistent with the TA spectra presented in Figure 6, where at low pH (6.0) a subtle transient feature appears in the low-energy region (620 nm), suggesting that MBV is populated alongside PCB_82_ from DBV, as reflected in our rate model. However, as explained earlier, this can only occur on ultrafast time scales, since vibrational relaxation can significantly alter the excitation energy landscape. Thus, one see the final populations of the sites does not follow the vertical excitation energies *E*_*m*_ but approximately the 0–0 transition energies *E*_0–0,*m*_ ^63,64^ which is the difference between the vertical excitation energy *E*_*m*_ and the associated reorganization energy of that pigment λ_*m*_, i.e., *E*_0–0,*m*_ = *E*_*m*_ - λ_*m*_. This point is important since the MBV molecules have quite different reorganization energies in their different protonation states. In the fully protonated system, the environmental effects on each bilin are similar, leading to nearly identical reorganization energies. In contrast, when the MBVs are deprotonated, the coupling to the intramolecular vibrational modes becomes stronger. This change makes the energy profiles of the 0–0 transition energies between the protonated and the deprotonated version of the PC645 complex look much more similar, as can be seen in Figure 10. In particular, the MBV pigments have the lowest energies in both cases, making them the terminal excitation sink in the complex, irrespective of their protonation state. In both protonation states, the MBV molecules are excitonically coupled to PCB_82_ and PCB_158_; however, the corresponding forward transfer rates are relatively slow, and the backward rates exceed the forward ones. This effect is especially pronounced in the deprotonated complex, where transfer from MBV to PCB_82_ is highly inefficient. Although MBV has a higher site energy than PCB_82_, the direction of energy flow is reversed due to its larger reorganization energy. As a result, the population on PCB_82_ eventually flows back to MBV, from where it may ultimately be released from the complex as heat. The exact details of this energy dissipation are, however, beyond the scope of the present study. Overall, the rate calculations indicate that in the protonated case, two pathways exist, with excitation transferring from DBV to either PCB_82_ or MBV. In contrast, in the deprotonated case, excitation reaches PCB82 only on short time scales; however, at longer time scales, all excitation ultimately accumulates on MBV in both the protonated and deprotonated cases, consistent with the *E*_0–0_ energy ladder.

**Figure 10:**
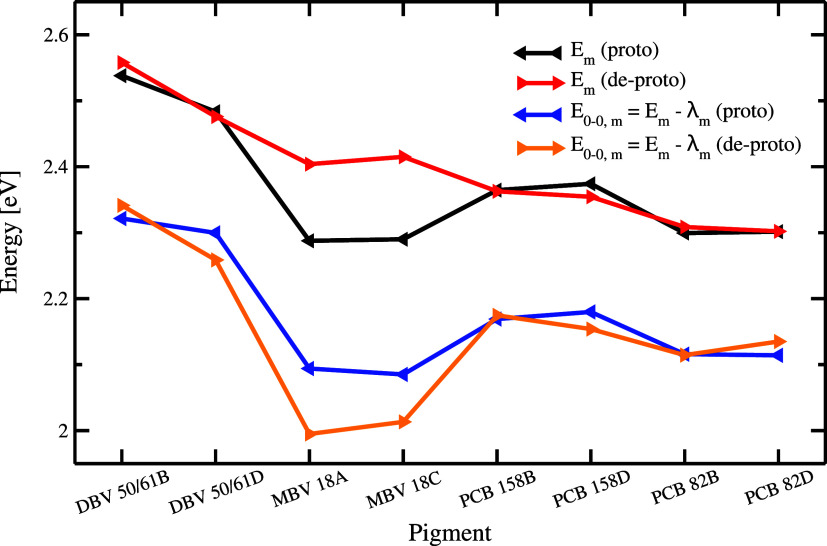
Comparison between vertical excitation energies *E*_*m*_ and 0–0 transition energies *E*_0–0,*m*_.

Importantly, the influence of reorganization energies becomes most clearly manifested in the long-time dynamics of the isolated PC645 complex, where vibrational relaxation is complete, and energy transfer follows the *E*_0–0_ energy landscape. In a biological context, however, where PC645 is coupled to the PSII antenna complex of cryptophyte algae, energy transfer may occur on time scales comparable to or even shorter than vibrational relaxation. Consequently, short-time population dynamics, which are strongly influenced by vertical excitation energies and electronic couplings, can deviate from long-time behavior, although reorganization effects are not entirely negligible.

Following the rate calculations, we computed the quantum yield. To do so, it is necessary to include, in addition to the Förster transfer rates, both radiative and nonradiative decay rates to the ground state in the rate matrix for each pigment, as indicated by the fluorescence experiments. However, atomistic calculations of these decay rates, particularly the nonradiative ones, are not straightforward for large molecules such as bilins, as many nonradiative processes, including intersystem crossing and internal conversion, involve complex excited-state dynamics, conical intersections, and spin–orbit coupling effects that are difficult to characterize accurately, especially in a biological environment.^65^ In addition, there are not many reports on the determination of pH-dependent radiative and nonradiative rates, especially for the present class of systems. Thus, each site is assigned both radiative and nonradiative decay rates based on experimental measurements, which are assumed to be intrinsic and independent of the environment. Under high-pH conditions, based on the *E*_0–0_ transition energies shown in Figure 10, MBV acts as the sole excitation sink, having the lowest energy among all bilins. Consequently, the decay rates of the PC645 complex are taken to correspond to those of MBV at high pH, as reported in Table 3. In the protonated case, however, all bilins are protonated, and although MBV still has the lowest *E*_0–0_, the energy gap relative to the other bilins, particularly PCB_82_ is smaller than in the deprotonated case. Therefore, we assume that all bilins in the protonated state share the same experimentally determined decay rates as those measured under low-pH conditions. Based on these decay rates, the population dynamics can be obtained from a classical rate model using

dP/dt=KP 9

where ***K*** is the extended Förster transfer rate matrix as given in Tables S8 and S9 in the SI, including radiative and nonradiative decay rates. The resulting dynamics are shown in Figure 11. The fluorescence quantum yield Φ represents the total probability of radiative decay, and is given by

$$\Phi =\int_{0}^{\infty }\sum_{m}k_{r,m}P_{m}(t)dt$$ 10

The resulting quantum yield of 25.8% at low pH is larger than the value of 15.1% at high pH, exactly reproducing experimental findings at pH 6.0 and 8.0 as given in Table 3, leading to a ∼40% quenching at high pH. The computed quantum yields use experimentally determined values for radiative and nonradiative decay rates. The absolute values of the quantum yields certainly strongly depend on these experimentally determined decay rates to the ground state. The present study, however, focused on the transfer rates between the excited states of the different pigments which were determined based on molecular simulations. We want to emphasize that these excited-state rates significantly influence the values for the quantum yields and are key in determining the difference between the quantum yields for the protonated and unprotonated complexes. The calculated and experimentally differences in quantum yield do nicely agree showing the power of the present strategy.

**Figure 11:**
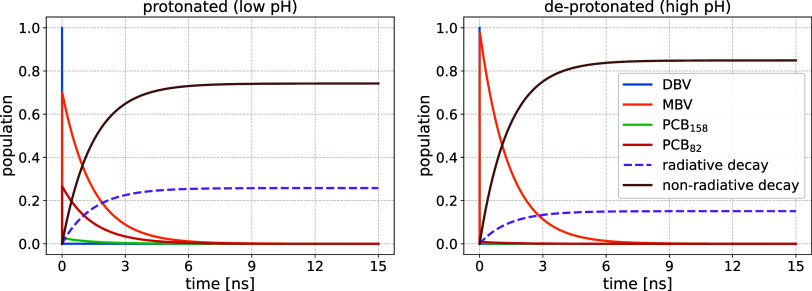
Population dynamics of the protonated and deprotonated systems. The purple dashed lines represent radiative decay, whose long-time limit reflects the fluorescence quantum yield, while the brown curves represent nonradiative decay channels.

It should be noted that the above conclusions rely on the assumption that all bilins are able to radiate. This assumption is based on the structural similarity of the bilins within the complex, which suggests similar decay pathways, although direct theoretical support is still lacking. Previous experimental work has reported that, at the red edge of the absorption spectrum, the emission anisotropy is just below 0.4, suggesting the presence of a single emitter in the PC645 energy cascade,^19^ although these measurements were performed at pH 7.5. While our results indicate that MBV has the lowest 0–0 transition energy and is ultimately populated, it cannot act as the sole fluorescent emitter, since it would lead to a quantum yield that is inconsistent with experimental observations. Moreover, the increase in quantum yield at low pH is consistent with the enhanced delocalization of the lowest excitonic states between MBV and PCB_82_ discussed above, which promotes the radiative rates. In contrast, at high pH, the increased reorganization energies of MBV pigments, also discussed above, likely enhance nonradiative rates, thereby accounting for the overall mechanism in the system.

### 2.8 Structural Similarity in Closed Phycobiliproteins

In order to identify a common adaptation involving an excitonic switch in cryptophyte algae, as outlined in the main text, we analyzed all the closed-form phycobiliprotein structures present in the Protein Data Bank. Apart from the PC645 complex (PDB: 4LMS),^12^ we also reviewed structures such as PC630 (PDB: 7T7U),^13^ PE545 (PDB: 7TJA),^66^ and PE566 (PDB: 7T8S).^13^ As shown in Figure 12, the bilin pigment within the α subunit consistently interacts with a crystallographic water molecule. Similar to the PC645 complex studied here, this bilin is termed mesobiliverdin (MBV) in the PC630 complex, whereas in the case of the PE545 and PE566 complexes, it is referred to as dihydrobiliverdin (DBV) and Bilin 618, respectively. Furthermore, previous pH-dependent absorption measurements of several cryptophyte phycobiliprotein complexes have shown that, among the closed-form systems, PC630 and PE545 exhibit pH-dependent absorption trends very similar to those of PC645, whereas the open-form PC577 displays a distinctly different behavior.^26^ These findings altogether further substantiate our hypothesis that closed-form phycobiliproteins feature a unique excitonic switch, mediated by the protonation state of a specific bilin pigment, which modulates the light-harvesting adaptation via its vibrational dynamics. Nonetheless, rigorous, protein-specific investigations will be required to confirm the universality of this mechanism.

**Figure 12:**
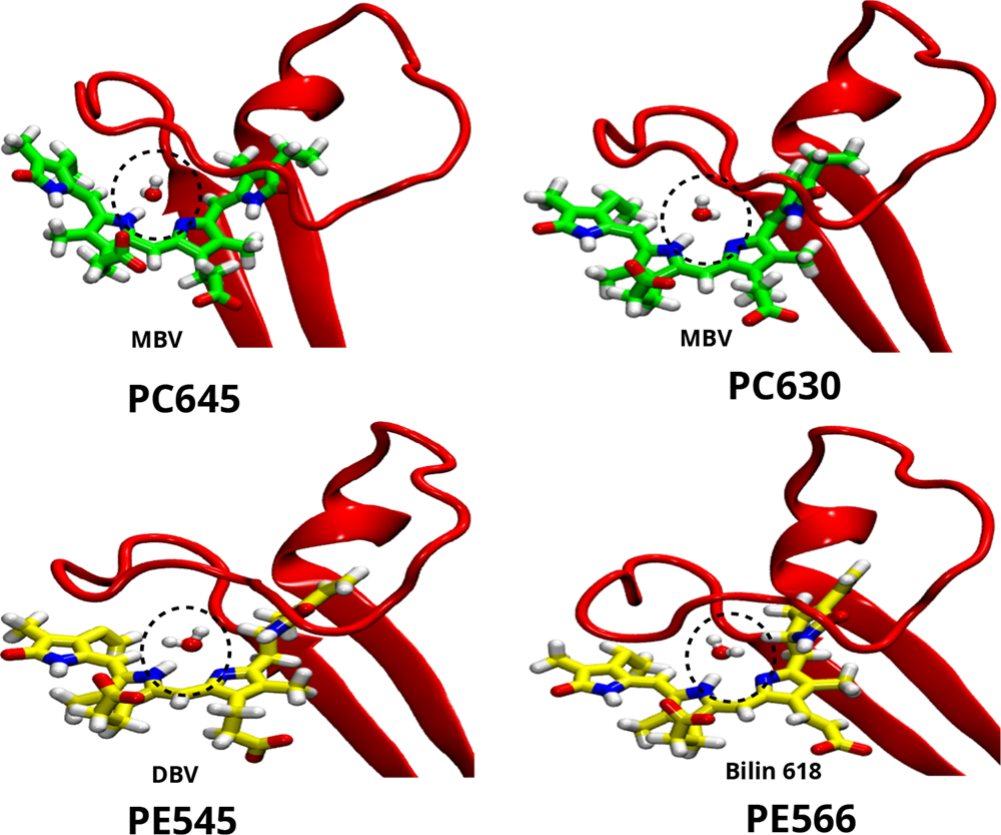
Structural comparison of closed-form phycobiliprotein complexes: PC645 and PC630 (upper panel) and PE545 and PE566 (lower panel) are available in the Protein Data Bank. The interaction of a specific bilin pigment with a crystallographic water molecule (highlighted by a dashed circle), as examined in this study for the PC645 complex, is also present in other complexes.

## 3 Conclusions and Outlook

In conclusion, this study provides new insight into the excitation energy transfer and quenching mechanisms in the cryptophyte PC645 complex, revealing previously unexplored aspects of photosynthetic regulation in this class of phycobiliproteins. By combining theoretical predictions with experimental validation, we demonstrate that the protonation states of the MBV bilins play a decisive role in governing the photophysics of the system. In contrast to earlier reports^9,19–22^ that identified PCB_82_ as the terminal emitter at least under neutral pH conditions, we find that MBV acts as the dominant terminal excitation sink across both low and high pH regimes, while PCB_82_ might as a terminal sink only in nonequilibrium regime at high pH (only based on the vertical excitation energies). Furthermore, protonation and deprotonation of the MBV pigments induce a coupled interplay between excitation energies, excitonic couplings, and intramolecular vibrations, leading to substantial variations in excitation energy transfer rates by up to a factor of two to three across different protonation states of the PC645 complex. This interplay might be part of a mechanism switching between light harvesting and energy dissipation of the complex at different pH conditions. Our rate model quantitatively reproduces the experimentally observed excited-state quenching at high pH, lending strong support to the robustness of this result. Notably, this quenching mechanism is fundamentally distinct from the so-called “non-photochemical quenching (NPQ)” in higher-plant membrane proteins, where a trans-thylakoid pH gradient induces quenching primarily through excitonic coupling between chlorophyll-carotenoid pairs, often modulated by protein conformational dynamics.^49,67–69^ Despite this clear mechanistic distinction, an open question remains regarding the physiological role of high-pH-induced quenching in PC645. In particular, it is not yet understood why such a pathway persists in the presence of phycobiliproteins on the lumenal side of the PSII complex in cryptophyte algae, nor how it influences energy transfer to the Chl-*a*/*c*-binding PSII antenna. This open question highlights an important gap in our current understanding. We therefore propose that this phycobiliprotein may function as an external regulatory sensor of the antenna system, analogous to PsbS in LHCII^70^ of higher plants and Lhcx1 in the FCP complexes of diatoms.^71^ This assumption is based on simulations showing that the FCP complex can be quenched at high pH, whereas physiological quenching at low lumenal pH occurs only when it binds to the Lhcx1 protein.^72^ At the same time, whether this functional analogy holds, and under which physiological conditions it becomes relevant, remains an open question. Furthermore, structural analysis of all available closed-form phycobiliproteins in the PDB database reveals a consistent presence of a specific bilin pigment that is accessible by water^13^ and therefore most likely undergoes protonation and deprotonation like the MBV_18_ in PC645 as studied here. This suggests a potentially conserved mechanism of quenching across closed-form phycobiliprotein structures, possibly mediated by pH-dependent modulation of this pigment. Such a conserved mechanism further shows the importance of the mechanism studied in this work, although its physiological function is not clear at the moment.

Overall, this study predicts a novel pH-dependent mechanism in closed-form phycobiliproteins using multiscale simulations, supported by steady-state and time-resolved experiments. Our findings provide a foundation for future research that may reshape our understanding of vibration-assisted photosynthetic regulation in cryptophytes and other natural light-harvesting systems, including plants, bacteria, and algae. Ultimately, this work challenges existing paradigms and opens new avenues for investigation, with potential implications for bioengineering and the development of advanced photosynthetic systems in both natural and synthetic contexts.

## 4 Computational Protocol

The initial structure of the PC645 complex used for the simulations was extracted from the high-resolution crystal structure reported by Harrop et al.^12^ (PDB IDs: 4LMS). The water-soluble system was modeled using the CHARMM-GUI Web server,^73^ with the Amber14SB force field applied to the protein and the GAFF2 force field to the bilin pigments. Initially, the system was prepared in the AMBER software file format. No missing residues in the protein chain were modeled. All amino acids were always assigned their standard protonation states as provided by CHARMM-GUI. The bilins were considered either protonated or deprotonated, depending on local protein interactions with the nitrogen atoms of the central pyrrole ring. Moreover, all bilins were covalently linked to the sulfur atoms of cysteine residues, and their partial charges were manually adjusted in the topology file. Additionally, the carboxylic groups of the bilins were treated as deprotonated, resulting in an overall charge of −1 for protonated bilins and −2 for deprotonated bilins. Finally, the AMBER files were converted to the file format of the GROMACS MD engine^74^ using the ACPYPE (AnteChamber PYthon Parser interfacE) tool,^75^ as GROMACS served as the primary simulation engine in this study. After system preparation, the system was equilibrated. A detailed equilibration procedure is outlined in the SI. The final structure after equilibration was utilized for subsequent DFTB-based QM/MM dynamics. We assumed uncorrelated fluctuations of the chromophores,^76,77^ treating them separately to obtain individual site energies. Each bilin was assigned to a QM region separately, with a cut at the C–S bond and hydrogen atom capping, while the rest of the system, including water, the protein, and the remaining seven bilins, was assigned to the MM region. The SCC-DFTB3 method^41^ with the 3OB-f parameter set^42^ was used for the QM region, while classical force fields were applied to the MM region. The 3OB-f parameter set provides a more accurate description of vibrational modes including C=C, C=N, and C=O bond stretching compared to the 3OB parameter set.^42^

A 100 ps QM/MM NPT equilibration was performed, followed by an additional 40 ps QM/MM MD simulation with a small integrator time step of 0.5 fs. The atomic coordinates were stored every 2 fs, resulting in 40,000 frames. These simulations were repeated three times in GROMACS with the DFTB+ interface,^51^ and the resulting data were used for the spectral density calculations. Furthermore, an extended simulation of 1 ns was carried out with a 1 fs time step after the initial 100 ps NPT equilibration, and the coordinates were saved every 100 fs, yielding 10,000 frames. The trajectories from these QM/MM ground state simulations were subjected to excited state calculations using the TD-LC-DFTB method^43^ using the DFTB+ 21.1 package.^78^ Autocorrelation functions were then determined from the site energy fluctuations, and spectral densities were computed via a cosine transformation.^34^ Additionally, we performed the same QM/MM MD simulations followed by TC-LC-DFTB calculations on two additional sets of initial geometries, extracted from a 200 ns classical MD trajectory at 100 ns intervals. The spectral densities of the individual pigments were then averaged across the three simulation sets.

Furthermore, to compute the excitonic couplings within the pigment network, we extended the classical MD simulation from 200 ns to 1 μs, extracting 100,000 snapshots from the full trajectory. After that, the TrESP charges were considered to calculate the couplings along the trajectory. Finally, time-averaged couplings were coupled together with time-averaged site energies to construct the average excitonic Hamiltonian as discussed earlier. The TrESP charges were computed based on TD-DFT (CAM-B3LYP/Def2-TZVP) calculations (especially within the Tamm–Dancoff approximation) using the ORCA quantum chemistry code,^79^ followed by the Multiwfn package.^80^ This procedure, previously used for Chl molecules,^81^ has been followed here. However, in this study, we consider the DFTB/MM optimized structures of the individual pigments and performed TrESP charge calculations while accounting for the surrounding point charge environment. This approach offers a more accurate representation of polarization by the external environment and is important for accurately evaluating excitonic coupling.^82,83^ Furthermore, the same TD-DFT calculations were applied to obtain excitation energies from the DFTB/MM optimized geometries, serving to benchmark the TD-LC-DFTB method, as discussed in the Supporting Information.

## 5 Experimental Methods

### 5.1 Sample Preparation

PC645 was harvested from *Chroomonas mesostigmatica* (Bigelow National Center for Marine Algae and Microbiota) grown in Prov50 media at room temperature under a 12 h on/off light cycle. Following growth to saturation after 2 weeks, algae were harvested using centrifugation (2000*g*) for 2 min, followed by resuspension of the pellet in 100 mM phosphate buffer (pH 7.2) and immediate freezing at −20 °C. After at least 1 day of freezing, the cells were thawed in the dark at room temperature to release proteins. Phycobiliproteins were then purified using ammonia sulfate precipitation, as previously reported.^25^ Phosphate buffer solutions (50 mM) were prepared by mixing appropriate proportions of sodium dihydrogen phosphate (NaH_2_PO_4_, monobasic) and disodium hydrogen phosphate (NaH_2_PO_4_, dibasic) in ultrapure water. The pH of each buffer was adjusted by the addition of small volumes of standardized sodium hydroxide (NaOH) or hydrochloric acid (HCl). Final pH values of 5.0, 5.5, 6.0, 6.5, 7.0, 7.5, and 8.0 were obtained with an uncertainty of ±0.05 pH units, as measured using a FisherbrandaccumetBasic AB315 benchtop pH/mV meter equipped with a calibrated glass electrode. Buffer solutions were prepared fresh prior to use. Purified phycobiliproteins were dialyzed overnight at 4 °C against the phosphate buffer solutions at the indicated pH values.

### 5.2 Protein Stability

Sodium dodecyl sulfate–polyacrylamide gel electrophoresis (SDS–PAGE) was used to assess purified phycobiliprotein integrity and to confirm that exposure to different pH conditions did not result in truncation or degradation. Proteins dialyzed into phosphate buffer at pH 6.0, 7.0, and 8.0 were analyzed using a Bio-Rad Mini-PROTEAN Tetra Vertical Electrophoresis Cell with 10% Mini-PROTEAN TGXprecast gels (10-well, 30 μL). Protein samples were prepared by mixing the protein at a 1:1 ratio with sample buffer (62.5 mM Tris-HCl, pH 6.8, 2% SDS, 25% (v/v) glycerol, 0.01% bromophenol blue, 5% β-mercaptoethanol) to denature and charge neutralize the protein. Gels were stained with AcquaStain Protein Gel Stain (Bulldog-Bio). Native gel electrophoresis was used to determine whether PC645’s oligomerized as a result of pH. Native gels were run using a Bio-Rad Mini-PROTEAN Tetra Vertical Electrophoresis Cell with 10% Mini-PROTEAN TGXprecast gels (10-well, 30 μL) in a running buffer of 25 mM Tris, 192 mM glycine. Protein samples were prepared by mixing the protein at a 1:1 ratio with sample buffer (62.5 mM Tris-HCl, pH 6.8, 40% (w/v) glycerol, 0.01% (w/v) bromophenol blue) to avoid denaturing and charge neutralizing the protein, which would disrupt changes in oligomerization.

### 5.3 Steady-State Measurements

UV–visible absorption spectra of PC645 at different pH levels were collected on an Agilent Cary 100 spectrophotometer. Excitation and photoluminescence spectra were collected on QuantaMaster (Horiba) with an integrating sphere at a right-angle geometry between the excitation and emission arms. A 10 mm path length quartz micro fluorometer cell was used for all measurements.

### 5.4 Transient Absorption

Narrowband transient absorption (TA) measurements were performed on a regenerative Ti:sapphire amplifier laser system with a 1 kHz repetition rate (Coherent Astrella). Pulses were delivered with a temporal width of ∼45 fs and a center wavelength of 800 nm. The output was separated by a beamsplitter to generate the pump and probe pulses. The pump pulses passed through a mechanical chopper that blocked every other pump pulse. An optical parametric amplifier (Light Conversion OperA, OPA) was used to generate narrowband pump pulses at 560 nm, measured by the transient absorption spectrometer (Ultrafast Systems Helios). Broadband probe pulses in the visible region were produced from supercontinuum generation with 800 nm radiation. All measurements were taken with the pump at the magic angle (54.7°) to the probe. An automated translation stage was used to introduce a time delay between the pump and the probe pulses. The two beams overlapped spatially and temporally onto a 1 mm quartz flow cell. The probe spectra for each pump-on and pump-off signal were detected by a built-in camera after the sample, recorded as the differential absorption spectra (ΔA). The reference spectrum was collected from a reflected portion of the probe beam captured by a second camera before the sample. During the measurements, samples were stored on ice, and a peristaltic pump set to a flow rate of 0.5 mL/min was used to flow the sample with an in-line syringe filter to prevent photodegradation and aggregation. The TA spectrum at each delay time was averaged for 1 s.

### 5.5 Time-Correlated Single Photon Counting (TCSPC)

Time-resolved fluorescence measurements were performed using a DeltaFlex TCSPC instrument (HORIBA Scientific). A diode laser was used to excite each sample at 507 nm (DeltaDiode, DD-510L). The emitted photons were detected at 665 nm. A regular quartz cuvette with a 10 mm path length was used. To correct the instrument response function (IRF), an aqueous Ludox colloidal silica solution was used, and a neutral density filter was placed before the detector to reduce excitation scatter while keeping all settings equivalent to those used for data collection of the samples. All fluorescence decays were deconvolved with the IRF and analyzed using the DeltaFlex software package.

### 5.6 Quantum Yield Measurements

Absolute quantum yield measurements were conducted on a QuantaMaster (Horiba) with an integrating sphere. A micro fluorometer cell with a 10 mm path length and 2 mm interior width was used. Each phycobiliprotein solution was diluted to an optical density below 0.05, as measured with the 2 mm path length oriented toward the excitation beam in the absorption spectrophotometer. For all quantum yield measurements, the cuvette was oriented such that the 10 mm path length faced the excitation port, while the 2 mm width faced the emission port. This geometry allowed sufficient protein concentration while minimizing inner filter effects and potential protein dissociation.

Scattering spectra of each sample and corresponding buffer blank from 540 to 580 nm across the excitation wavelength at 560 nm were collected. Absorbed photons were calculated by subtracting the integrated scattering signal of the sample from the blank. Emission spectra of each sample and blank from 600 to 800 nm were collected to measure the number of emitted photons, which were calculated as the difference between the integrated emission of the sample and the blank. The absolute quantum yield for each sample was determined from the ratio of emitted photons to absorbed photons. The standard deviations of the mean quantum yields were calculated from five independently prepared replicate measurements. The step size was set to 0.25 nm for all measurements.

## Supplementary Material



## Data Availability

Computational data are publicly available on Zenodo (https://zenodo.org/records/19688206), including system equilibration files, QM/MM and classical MD trajectories, as well as site (excitation) energies, spectral densities, and TrESP charges of pigment molecules for both protonated and deprotonated states.

## Notes

Details on the equilibration procedure, excitation energy ladder, excitonic couplings, transition dipoles, reorganization energies, and additional experimental information regarding protein integrity at different pH and quantum yields (PDF)
