# Supplementary material for: pH-Dependent Vibrational Dynamics Drives Excited-State Quenching in the Phycobiliprotein Complex PC645
Source: J Am Chem Soc. 2026 Jul 3;148(27):29107–23. doi: 10.1021/jacs.6c07743 (PMC13383742; doi:10.1021/jacs.6c07743)
Supplement: Supplementary file 1 [file ja6c07743_si_001.pdf]

SUPPLEMENTAL INFORMATION
pH-Dependent Vibrational Dynamics Drives Excited-State Quenching in the Phycobiliprotein Complex PC645

Sayan Maity[1],[*,†,‡] Yue Hu[1],[¶] Dongyu Lyu[1],[†] Jingyun Wu,[¶] Coral O'Brien,[§] Michael H. Hecht,[¶] Leah C. Spangler,[‖] Gregory D. Scholes,[*,¶] and Ulrich Kleinekathöfer[*,†]

†*School of Science, Constructor University, Campus Ring 1, 28759 Bremen, Germany*

‡*Department of Physics and Astronomy and Thomas Young Centre, University College London, London WC1E 6BT, U.K.*

¶*Department of Chemistry, Princeton University, Princeton, New Jersey 08544, United States*

§*Center for Pharmaceutical Engineering and Sciences, Virginia Commonwealth University, Richmond, Virginia 23284, United States*

‖*Department of Chemical and Life Science Engineering, Virginia Commonwealth University, Richmond, Virginia 23284, United States*

E-mail: smaity@constructor.university; gscholes@princeton.edu; ukleinekathoefer@constructor.university

[1] SM, YH, and DL contributed equally to this work.

S1 Equilibration Procedure

After preparing the system, energy minimization was conducted to eliminate any bad contacts. Subsequently, a 2 ns NVT equilibration was carried out at 300 K, with a 1 fs integration time step to heat the system. This was followed by four consecutive 5 ns NPT equilibration steps, each utilizing a 1 fs time step. During these equilibration stages, position restraints were gradually applied to different components of the system: first on both the protein and pigment molecules, then only on the protein, next on the protein backbone, and finally only on the C_α atoms of the protein. After this, a 10 ns NPT equilibration was conducted without any restraints, also using a 1 fs time step. To further stabilize the system, another 500 ns NPT equilibration was performed with a 2 fs integration time step. Hence, a total of 530 ns long NPT equilibration was performed for the PC645 complex. Temperature and pressure were regulated using the Nose-Hoover thermostat[1] and the Parrinello-Rahman barostat[2], respectively. Short-range non-bonded interactions were computed using a 1.2 nm cutoff, while long-range electrostatic interactions were handled with the Particle Mesh Ewald (PME) method[3]. Moreover, the LINCS algorithm[4] was employed to maintain bond constraints and periodic boundary conditions (PBC) were consistently applied throughout all simulations.

S2 Benchmark of Excitation Energy Landscape

In the main text, we compared the average excitation energies of individual pigments computed at the TD-LC-DFTB level along a 1 ns QM/MM MD trajectory with previously reported values. Here, we benchmark the TD-LC-DFTB method against TD-LC-DFT to validate the excitation energy hierarchy presented in the main text. Specifically, we calculated excitation energies at the TD-DFT (CAM-B3LYP/Def2-TZVP) level using QM/MM-optimized geometries obtained at the DFTB level within a QM/MM framework. The same calculations were then repeated using the TD-LC-DFTB method, and the results are shown

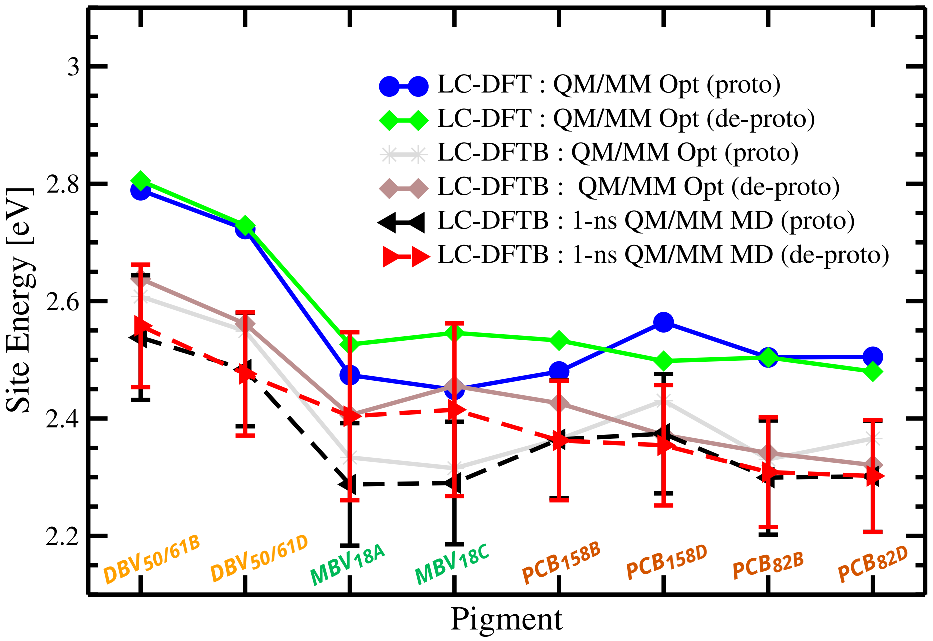

Figure S1: Benchmark of site energies for each pigment relative to the TD-DFT level of theory based on QM/MM optimized structures. Additionally, site energies along 1 ns QM/MM MD trajectories together with the error bar are also provided.

in Fig. S1. As evident from the figure, both TD-LC-DFT and TD-LC-DFTB methods exhibit the same trend in the site energy hierarchy, although the TD-LC-DFT values are slightly elevated, consistent with previous findings for BChl and Chl pigments in bacteria[5] and algae[6]. Additionally, we present the average excitation energies in the same Fig. S1 computed using TD-LC-DFTB along the 1 ns QM/MM MD trajectory as shown in the main text, which fall within a similar range as those obtained from QM/MM-optimized structures. This benchmark suggests that TD-LC-DFTB should be used for trajectory analysis for bilin pigments as well as similar light-harvesting complexes of plants, bacteria, and algae[6,7].

S3 Density of States

Next, we carried out TD-LC-DFTB calculations on the trajectories and examined the excitation energy distributions over the 1 ns and the initial 40 ps QM/MM MD trajectories, which are commonly referred to as the density of states (dos). The results, depicted in Fig. S2,

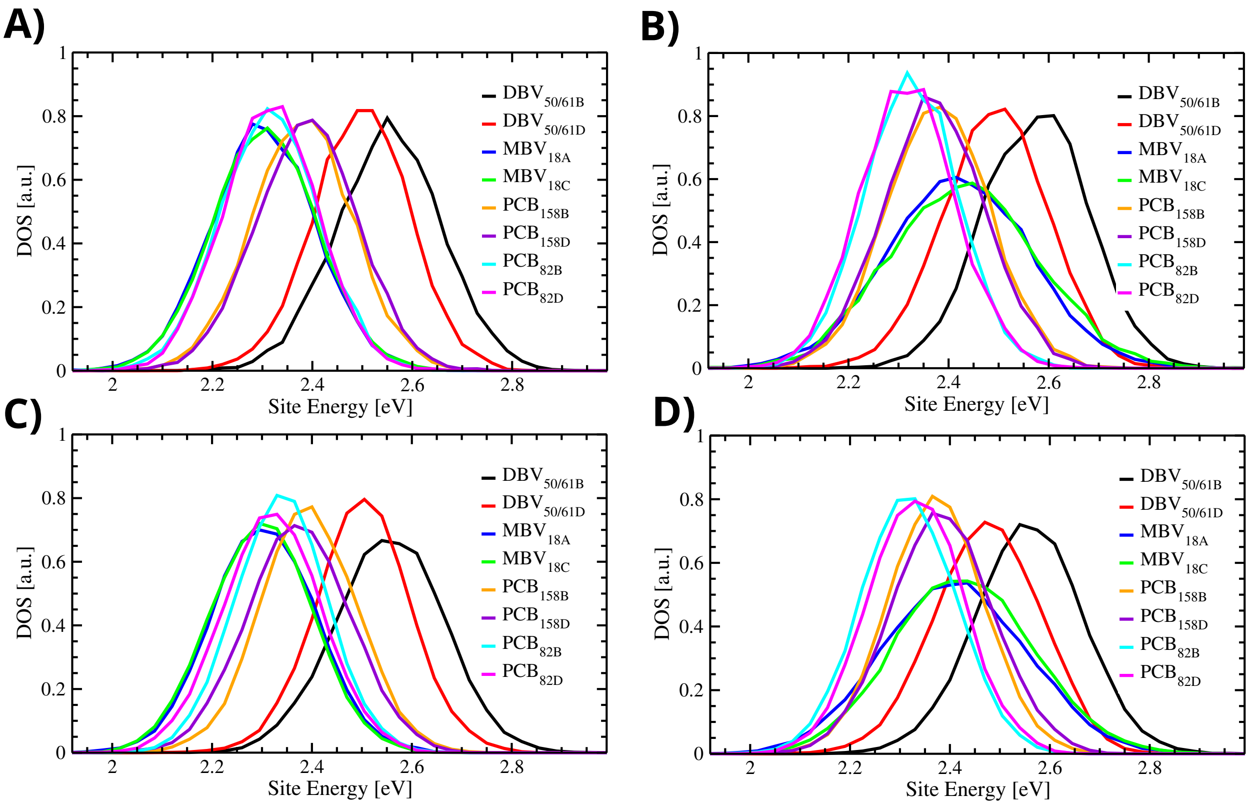

Figure S2: The site energy distribution of each pigment, also referred to as the density of states, is presented for the protonated states in (A) and the deprotonated states in (B) along 1 ns QM/MM MD trajectories. Similarly, (C) and (D) represent the same as (A) and (B), but for the first set of 40 ps QM/MM MD trajectories.

are provided for both the protonated and deprotonated states. The distributions exhibit a Gaussian shape, consistent with previous findings for BChl and Chl pigments in bacteria and plants using the TD-LC-DFTB method. In both the 1 ns and 40 ps trajectories, a similar trend is observed: $DBV_{50/61}$ consistently exhibits higher site energies. However, in the protonated state, MBV_{19} displays lower site energies, whereas in the deprotonated state, PCB_{82} acts as the terminal emitter.

S4 Excitonic Couplings and Hamiltonian

The excitonic coupling values between all pigment pairs were computed based on the so-called TrESP approach (Transition Charge from Electrostatic Potential)[8]. The scheme has

been widely recognized for being accurate for medium and large distances. Once the atomic transition charges of the pigments are determined, the coupling values can be written as

$$V_{mn} = \frac{f}{4\pi\epsilon_0} \sum_{I\in m} \sum_{J\in n} \frac{q_I^T \cdot q_J^T}{|\mathbf{r}_m^I - \mathbf{r}_n^J|} \ , \tag{1}$$

where q_I^T and q_J^T denote the transition charges of atoms I and J, and f is a screening factor taking environmental influences on the excitonic coupling into account. And in the present study, we have used a constant screening factor of 0.69, which is recommended for light-harvesting complexes[9]. The TrESP charges were calculated at the CAM-B3LYP/Def2-TZVP level of theory (see more details in the **Computational Protocol** section in the main text).

Table S1: Average excitonic Hamiltonian of the PC645 complex. The excitonic couplings for the protonated and deprotonated systems are shown in the upper and lower triangles, respectively. The site energies on the diagonal correspond to the protonated pigments, extracted from 1 ns QM/MM MD trajectories. All energies are in cm^{-1}. Couplings with absolute values greater than 50 cm^{-1} are highlighted in **bold**.

Bilins	$DBV_{50/61B}$	$DBV_{50/61D}$	MBV_{18A}	MBV_{18C}	PCB_{158B}	PCB_{158D}	PCB_{82B}	PCB_{82D}
$DBV_{50/61B}$	20469	**477.16**	14.47	**-60.16**	30.33	41.43	**-60.81**	27.04
$DBV_{50/61D}$	**444.78**	20029	**-64.04**	10.40	35.71	40.65	30.09	**-67.94**
MBV_{18A}	12.23	**-56.19**	18451	-5.30	**119.33**	3.87	18.99	**74.30**
MBV_{18C}	**-52.99**	11.21	-4.65	18471	3.15	**122.36**	**73.08**	17.36
PCB_{158B}	27.79	32.41	**105.99**	2.84	19071	12.73	16.30	-13.05
PCB_{158D}	37.88	37.46	3.40	**112.97**	12.97	19148	-12.30	15.67
PCB_{82B}	**-56.87**	29.41	16.33	**64.62**	14.15	-12.66	18546	-8.26
PCB_{82D}	26.73	**-64.14**	**66.85**	16.86	-13.29	16.82	-7.58	18563

The average couplings for the protonated and deprotonated states are shown in Table S1, together with the site energies obtained from 1 ns QM/MM trajectories at the protonated state, which are used as diagonal elements to construct the excitonic Hamiltonian of the system. A similar matrix, using site energies from the deprotonated state, is provided in Table S2 of the SI. As demonstrated in previous studies, here we also found that the strongest coupling contributions are observed for the $DBV_{50/61B}$/$DBV_{50/61D}$ pair with coupling values exceed-

ing 450 cm^{-1}. In addition, the DBV bilins show moderate coupling values greater than 50 cm^{-1} with nearby MBV and PCB pigments, which vary depending on the intermolecular distances. These pairs include $DBV_{50/61B}/MBV_{18C}$, $DBV_{50/61B}/PCB_{82B}$, $DBV_{50/61D}/MBV_{18A}$, and $DBV_{50/61D}/PCB_{82D}$. Moreover, the MBV_{18A}/PCB_{82D} and MBV_{18C}/PCB_{82B} pairs also exhibit moderate coupling values in the same range, indicating that excitons can be transferred from $DBV_{50/61}$ to PCB_{82} via MBV_{18}. Moreover, the upper panel of Fig. S3 shows the distribution of strong and moderate coupling pairs, while the lower panel presents a zoomed-in view highlighting the strong couplings between DBV/DBV and DBV/MBV at different protonation states. These couplings play a key role in governing the exciton transfer pathways within the pigment network. Experimental results have confirmed that excitation energy is transferred from the highest energy DBV pairs to the terminal emitter PCB_{82} [10]. However, since the coupling between these two pairs is not as strong as observed in our study, it has been reported that intramolecular vibrations play a significant role in this transfer process. Our calculations further show that when the MBV bilins are deprotonated, PCB_{82} serves as the terminal emitter. In contrast, when the MBV bilins are protonated, their excitation energies decrease significantly, causing them to act as energy sinks. Additionally, notably strong couplings greater than 100 cm^{-1} are found between MBV_{18A}/PCB_{158B} and MBV_{18C}/PCB_{158D}. Thus, while trapping excitons in their protonated state, MBV_{18A} and MBV_{18C} can transfer these excitations to PCB_{158B} and PCB_{158D} in the deprotonated state.

Table S2: Same as Table S1, except the diagonal elements have been replaced with site energies of the bilin pigments in the deprotonated state.

Bilins	$DBV_{50/61B}$	$DBV_{50/61D}$	MBV_{18A}	MBV_{18C}	PCB_{158B}	PCB_{158D}	PCB_{82B}	PCB_{82D}
$DBV_{50/61B}$	20631	**477.16**	14.47	**-60.16**	30.33	41.43	**-60.81**	27.04
$DBV_{50/61D}$	**444.78**	19970	**-64.04**	10.40	35.71	40.65	30.09	**-67.94**
MBV_{18A}	12.23	**-56.19**	19388	-5.30	**119.33**	3.87	18.99	**74.30**
MBV_{18C}	**-52.99**	11.21	-4.65	19478	3.15	**122.36**	**73.08**	17.36
PCB_{158B}	27.79	32.41	**105.99**	2.84	19056	12.73	16.30	-13.05
PCB_{158D}	37.88	37.46	3.40	**112.97**	12.97	18989	-12.30	15.67
PCB_{82B}	**-56.87**	29.41	16.33	**64.62**	14.15	-12.66	18620	-8.26
PCB_{82D}	26.73	**-64.14**	**66.85**	16.86	-13.29	16.82	-7.58	18567

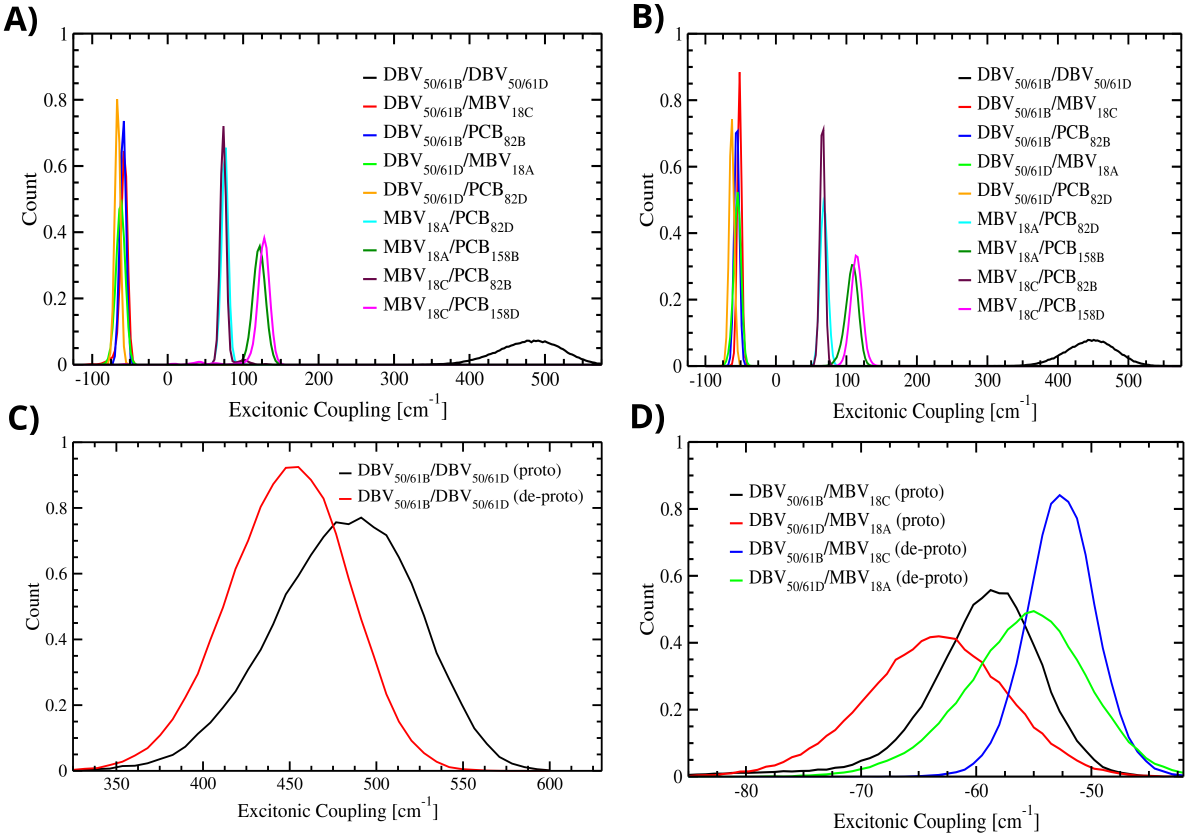

Figure S3: Distributions of strong excitonic couplings between various pigment pairs are shown for (A) the protonated state and (B) the deprotonated state. Additionally, enlarged views of the coupling distributions specifically for the DBV/DBV and DBV/MBV pairs at different protonation states are presented in (C) and (D), respectively.

Based on the excitonic Hamiltonians for the protonated and deprotonated states, presented in Table 1 of the main text and Table S2, we extracted the excitonic states in the PC645 complex. The corresponding excitonic energies are reported in the main text, while the contributions of individual pigments to each excitonic state, represented in the site basis, are detailed in Table S3 and Table S4 for the protonated and deprotonated states, respectively.

The highest excitonic states (states 7 and 8) are primarily contributed by DBV pigments in both protonation states. However, in the protonated state, the lowest excitonic states (states 1 and 2) are mainly composed of contributions from MBV pigments, while in the deprotonated state, the PCB_{82} bilins dominate the lowest excitonic states. This clearly indi-

Table S3: Site basis contributions of pigments to the excitonic states in the protonated PC645 complex. Pigment contributions to the highest and lowest excitonic states are highlighted in bold, with red and blue colors indicating the respective states.

	**Exciton 1**	**Exciton 2**	**Exciton 3**	**Exciton 4**	**Exciton 5**	**Exciton 6**	**Exciton 7**	**Exciton 8**
$DBV_{50/61B}$	0.00	-0.02	-0.06	-0.01	-0.01	0.00	**0.54**	**0.84**
$DBV_{50/61D}$	0.02	0.02	0.07	-0.01	-0.04	0.05	**-0.84**	**0.54**
MBV_{18A}	**0.73**	**0.53**	0.15	0.18	0.04	0.35	0.05	-0.01
MBV_{18C}	**0.47**	**-0.73**	-0.37	-0.02	0.17	0.27	-0.04	-0.02
PCB_{158B}	-0.13	-0.11	0.00	0.97	0.15	-0.09	-0.01	0.03
PCB_{158D}	-0.08	0.13	0.05	-0.15	0.97	-0.08	-0.03	0.03
PCB_{82B}	-0.31	0.35	-0.77	0.04	0.00	0.44	-0.05	-0.02
PCB_{82D}	-0.35	-0.18	0.49	0.00	0.04	0.77	0.06	-0.01

cates that exciton transfer proceeds from the highest-energy DBV states to the lowest-energy states, either MBV or PCB_{82}, depending on the protonation state of MBV, as discussed in detail in the main text.

Table S4: Same as Table S3 but at deprotonated state.

	**Exciton 1**	**Exciton 2**	**Exciton 3**	**Exciton 4**	**Exciton 5**	**Exciton 6**	**Exciton 7**	**Exciton 8**
$DBV_{50/61B}$	-0.02	0.04	-0.03	0.00	-0.07	0.08	**0.44**	**0.89**
$DBV_{50/61D}$	0.05	-0.04	-0.01	0.06	0.14	-0.11	**-0.87**	**0.45**
MBV_{18A}	-0.08	-0.01	0.01	0.28	0.94	0.09	0.15	-0.01
MBV_{18C}	-0.02	-0.08	-0.21	0.04	-0.08	0.96	-0.14	-0.03
PCB_{158B}	0.04	-0.03	-0.12	-0.95	0.28	0.03	0.00	0.02
PCB_{158D}	-0.03	0.06	0.97	-0.11	0.00	0.21	-0.04	0.03
PCB_{82B}	**0.11**	**0.99**	-0.08	-0.01	0.03	0.06	-0.05	-0.02
PCB_{82D}	**0.99**	**-0.11**	0.04	0.06	0.05	0.04	0.06	0.00

S5 Transition Dipole Moment

The transition dipole moment is a crucial property to model excited-state processes, directly influencing excitonic coupling and absorption spectrum. Since transition charges in the TrESP Coulomb couplings essentially represent the transition dipole moment of the molecule, variations in coupling can be observed across different protonation states due to this property. As shown in Table S5, the transition dipole moment exhibits a slightly higher value in the protonated state, particularly for MBV_{19} bilins, which is also reflected in the coupling distributions involving this pigment shown in the main text. However, when considering overall rate changes, spectral density and reorganization energies emerge as the key properties

highlighted in the main text.

Table S5: Magnitudes of the transition dipole moments (in Debye) for each bilin pigment in both protonated and deprotonated forms. MBV values are highlighted in boldface.

**Bilins**	**protonated PC645**	**deprotonated PC645**
$DBV_{50/60B}$	11.94	11.87
$DBV_{50/60D}$	12.33	12.26
MBV_{18A}	**13.24**	**12.33**
MBV_{18C}	**13.47**	**12.19**
PCB_{158B}	13.03	13.06
PCB_{158D}	13.02	13.21
PCB_{82B}	13.16	13.22
PCB_{82D}	13.16	13.00

S6 Average Spectral Density

In this section, the average spectral density of the PC645 complex at different protonation states is compared and shown in Fig. S4, alongside the spectral density calculated by Blau et al.[11]. As depicted in the figure, the deprotonated spectral density exhibits more high-frequency vibrational peaks compared to the protonated state, which is primarily attributed to the protonation and deprotonation of the MBV_{19} bilins, as discussed in the main text. Additionally, the spectral density calculated by Blau et al. also shows more high-frequency peaks. Although they did not specify the protonation states of the bilin molecules, it is expected that all pigments were considered deprotonated in their study. Furthermore, the high-frequency peaks in their calculation are shifted due to the use of the B3LYP functional for the QM/MM MD simulations. This functional is known to overestimate vibrations of C=C, C=N, and C=O bonds compared to the vibrationally corrected DFTB parameter 3OB-f set, as identified in a previous study[12].

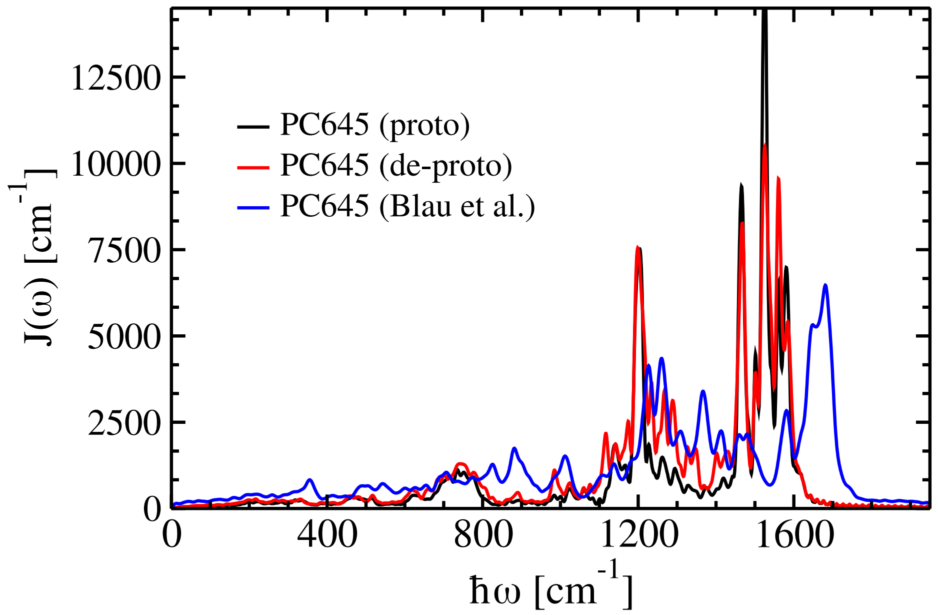

Figure S4: Average spectral density at protonated and de-protonated states in comparison with Blau et al.[13]

S7 Reorganization Energy

The reorganization energy can be directly extracted from the spectral density by

$$\lambda_m = \int_0^\infty \frac{J_m(\omega)}{\omega} d\omega \ . \tag{2}$$

Table S6: Reorganization energies (λ in cm^{-1}) of the bilin pigments for the protonated and deprotonated PC645 complexes together with the average values. The λ values for the MBV pigments are given in boldface to highlight the significant changes in these values.

**Pigment**	**Proto (λ cm^{-1})**	**De-proto (λ cm^{-1})**
$DBV_{50/61B}$	1745	1747
$DBV_{50/61D}$	1481	1753
MBV_{18A}	**1561**	**3298**
MBV_{18C}	**1653**	**3240**
PCB_{158B}	1576	1516
PCB_{158D}	1566	1617
PCB_{82B}	1480	1566
PCB_{82D}	1511	1347
Average	1572	2011

The extracted reorganization energies for the protonated and deprotonated states are

summarized in Table S6. From atomistic spectral densities, the average reorganization energies are calculated as 1572 cm^{-1} and 2011 cm^{-1} for the two states, respectively. In comparison, Blau et al.[11] reported an average reorganization energy of 909 cm^{-1}. Earlier studies, however, reported significantly lower reorganization energies in both computational and experimental work[14,15], where only a single low-frequency peak or one low-frequency and one high-frequency peak were considered. Our calculations, as well as the findings by Blau et al.[11], clearly indicate that spectral densities are characterized by multiple peaks, leading to higher reorganization energies. Furthermore, the table shows that the reorganization energies in deprotonated MBV are more than twice as high as those in the protonated structure. This is because of multiple high-frequency peaks present in the spectral densities of deprotonated MBVs.

S8 Protein Integrity under Varying pH

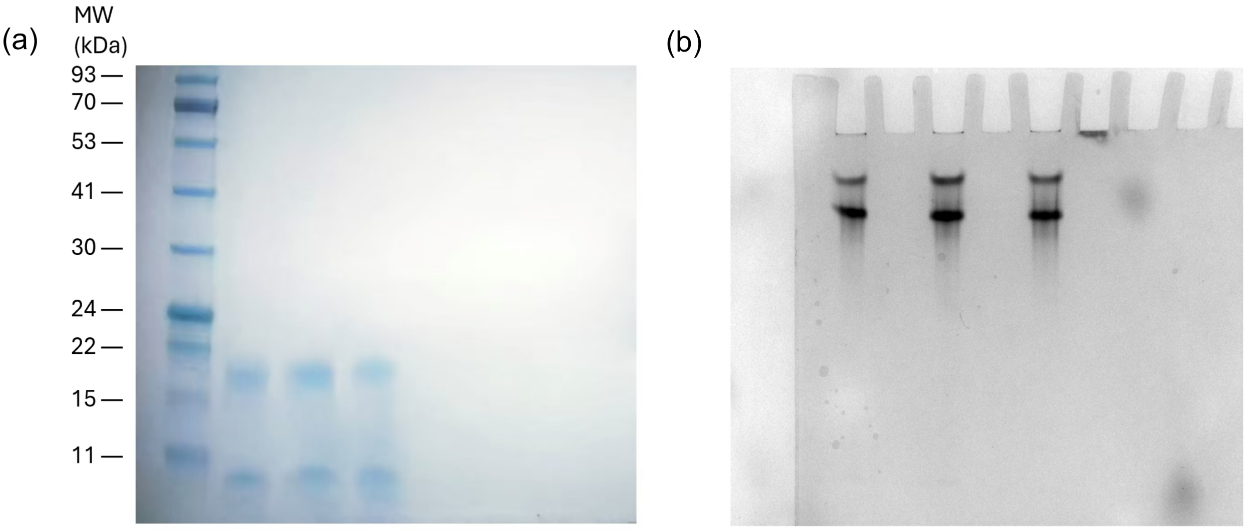

Figure S5: (a) SDS-PAGE and (b) native gel electrophoresis results of PC645 at pH levels of 6.0, 7.0, and 8.0. Panel (a) shows one band (~20 kDa) that corresponds to one β subunit, and the other band (~10 kDa) that corresponds to one α subunit.

To ensure that observed changes in optical properties were not due to alterations in protein integrity, PC645 samples were subjected to electrophoretic analysis following dialysis

at pH 6.0, 7.0, and 8.0. Samples were analyzed by SDS-PAGE under denaturing conditions and by native gel electrophoresis. Fig. S5(a) shows two bands corresponding to the α (~10 kDa) and β (~20 kDa) subunits, with no additional bands observed across pHs, indicating preservation of protein integrity and purity. Native gel analysis in panel (b) similarly reveals consistent band patterns at all pH values, indicating PC645 remains unchanged. In addition, transient absorption spectra at long delay times show no pH-dependent shifts in peak positions (Fig. S6), and emission maximum remains constant with pH (Fig. 6(b)). These results confirm that the protein complex remains structurally intact over the pH range studied, and the photophysical changes are therefore not a result of protein oligomerization or fragmentation.

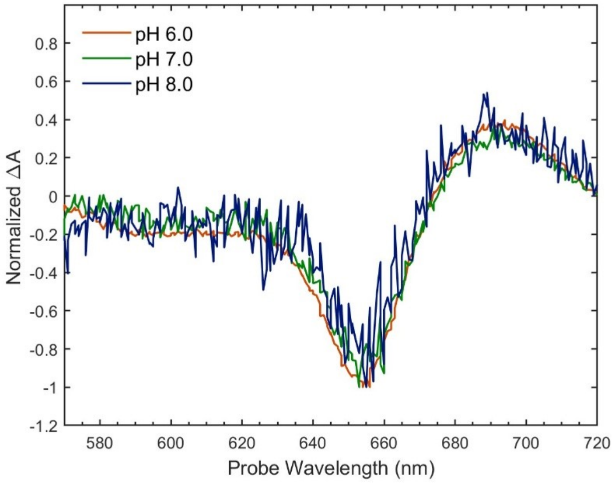

Figure S6: Normalized TA spectra of PC645 at 1 ns in phosphate buffer at pH levels of 6.0, 7.0, and 8.0 when excited at 560 nm. Peak positions remain unchanged at varying pH values, showcasing the structural integrity of the protein.

S9 Fluorescence Quantum Yield

We measured the fluorescence quantum yield 5 times at each pH level, and the results of each trial are shown in Table S7.

Table S7: Quantum yields and their associated standard deviations for PC645 in phosphate buffer at varying pH levels.

**pH**	**Quantum Yield (%)**					**Average Quantum Yield (%)**	**Standard Deviation (%)**
	**Trial 1**	**Trial 2**	**Trial 3**	**Trial 4**	**Trial 5**		
**5.0**	23.1	36.6	32.2	28.1	25.1	29.0	5.5
**5.5**	28.0	26.5	24.6	29.3	23.3	26.3	2.4
**6.0**	24.8	29.9	23.4	27.8	23.0	25.8	3.0
**6.5**	23.9	24.8	27.1	23.7	20.8	24.1	2.3
**7.0**	21.6	19.5	18.0	17.5	20.0	19.3	1.6
**7.5**	18.9	16.6	20.6	18.3	19.5	18.8	1.5
**8.0**	14.3	15.8	13.5	15.1	16.2	15.0	1.1

S10 Efficiency of Excitation Absorbed by Different Bilins

To assess how efficiently excitation absorbed by different bilins contributes to the observed emission, excitation spectra were compared to absorption spectra for pH 6.0, 7.0, and 8.0 (Fig. S7). Absorption and excitation spectra were normalized at the high-energy DBV peak to highlight relative differences in the mid- and low-energy regions.

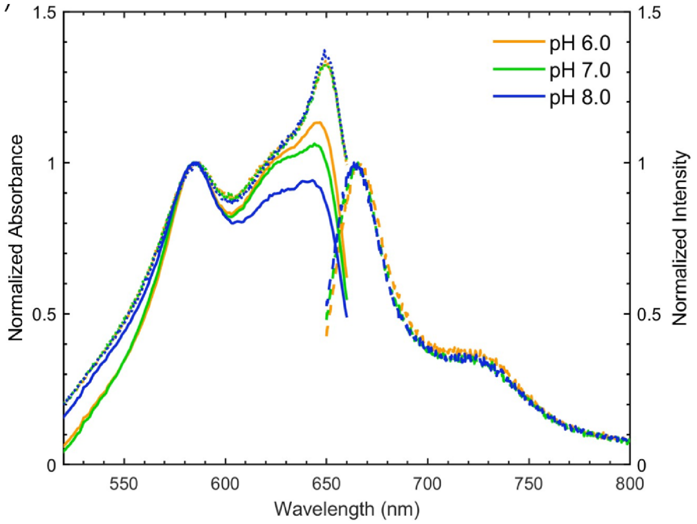

Figure S7: Absorbance spectra (solid lines), excitation spectra (dotted lines), and photoluminescence spectra (dashed lines) of PC645 at pH 6.0 (orange), pH 7.0 (green), and pH 8.0 (blue). Absorbance and excitation spectra are normalized at the high-energy peak to highlight spectral differences in the mid- and low-energy regions.

The excitation spectrum reflects how efficiently absorbed photons lead to emission. As shown in Fig. S7, differences between the absorption and excitation spectra indicate that nonradiative processes are active within the bilin network. At pH 6.0 and 7.0, the excitation spectra closely follow the absorption spectra and exhibit relatively high intensity in the low-energy region. In contrast, at pH 8.0, the excitation spectrum deviates more significantly from the absorption spectrum. These results imply that pH alters the relative efficiency of excitation pathways feeding the final emitting state. As discussed in the main text for the absorption spectra, the low-pH condition involves contributions from both MBV and PCB_{82}, whereas at high pH the signal is dominated by PCB_{82}. The excitation spectra at low pH further support the conclusion that MBV contributes to emission alongside PCB, consistent with our interpretation in the main text.

S11 Förster Transfer Rate Matrix

The complete Förster transfer rate matrices for protonated and de-protonated systems are shown in Table S8 and Table S9, where the off-diagonal elements k_{ij} refer to the transfer rates of $j \to i$, while the diagonal elements k_{ii} are defined as the negative sum of all rates out of pigment i.

Table S8: Transfer rate matrix (in ps^{-1}) of protonated PC645 complex.

Bilins	$DBV_{50/61}$	MBV_{18}	PCB_{158}	PCB_{82}
$DBV_{50/61}$	-4.2007	0.0005	0.0028	0.0010
MBV_{18}	1.7921	-0.5376	3.9474	1.0207
PCB_{158}	0.5223	0.1490	-3.9930	0.0046
PCB_{82}	1.8863	0.3881	0.0043	-1.0264

Table S9: Same as Table S8 but for the de-protonated PC645 complex.

Bilins	$DBV_{50/61}$	MBV_{18}	PCB_{158}	PCB_{82}
$DBV_{50/61}$	-2.5681	0.0000	0.0056	0.0060
MBV_{18}	0.7501	-0.0071	1.5169	0.4551
PCB_{158}	0.4183	0.0030	-1.5605	0.0083
PCB_{82}	1.3996	0.0041	0.0380	-0.4694

S12 Detailed Balance

As a validity test of the rate model used to perform population dynamics, we analyze the detailed balance of the final populations at thermal equilibrium based on the Boltzmann distribution. For a system with N sites and denoting the the minimum energy by $E_{0-0,m} = E_m - \lambda_m$ the population at site i is given by

$$P_i = \frac{\exp[-E_{0-0,i}/(k_B T)]}{\sum_{j=1}^{N} \exp[-E_{0-0,j}/(k_B T)]} \ . \quad (3)$$

We find that the Boltzmann distribution is satisfied at thermal equilibrium, where the final populations obtained from the population dynamics based on the rate matrix exactly match those predicted by the Boltzmann distribution (see Fig. S8). This further validates the use of Förster theory in the present study and the subsequent rate model.

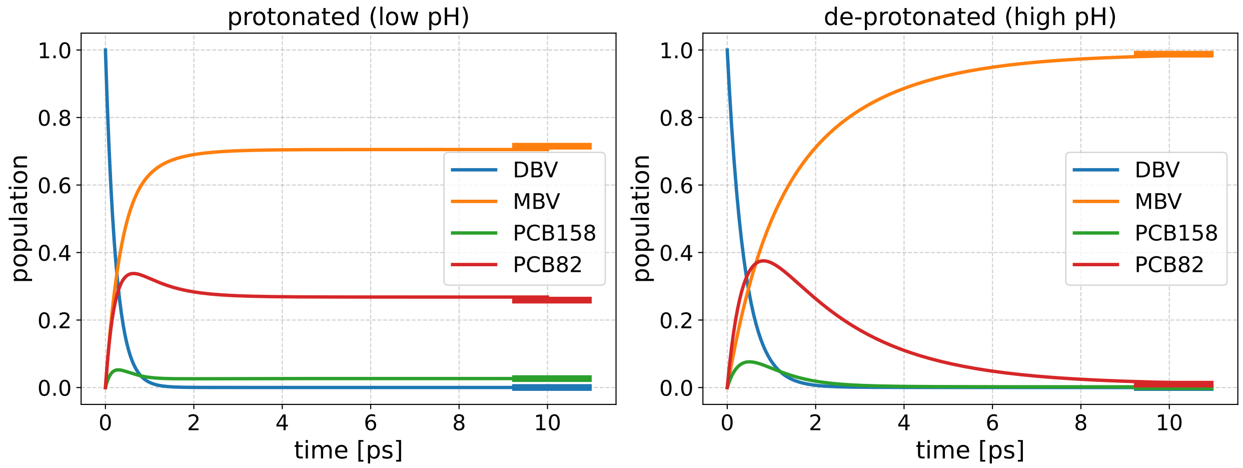

Figure S8: Thermal equilibrium populations predicted by the Boltzmann distribution as given by Eq. 3 are shown as horizontal bars at long times.

However, as one can see, this approach struggles to accurately predict the dynamics at short timescales, where the reorganization energy begins to dominate after 500-800 fs. For an improved discussion, for example, a non-equilibrium Förster theory can be applied[16,17].

References

(1) Evans, D. J.; Holian, B. L. The Nose–Hoover Thermostat. *J. Chem. Phys.* **1985**, *83*, 4069–4074, DOI: 10.1063/1.449071.

(2) Parrinello, M.; Rahman, A. Polymorphic Transitions in Single Crystals: A New Molecular Dynamics Method. *J. Appl. Phys.* **1981**, *52*, 7182–7190, DOI: 10.1063/1.328693.

(3) Essmann, U.; Perera, L.; Berkowitz, M. L.; Darden, T.; Lee, H.; Pedersen, L. G. A Smooth Particle Mesh Ewald Method. *J. Chem. Phys.* **1995**, *103*, 8577–8593, DOI: 10.1063/1.470117.

(4) Hess, B.; Bekker, H.; Berendsen, H. J. C.; Fraaije, J. G. E. M. LINCS: A Linear Constraint Solver for Molecular Simulations. *J. Comput. Chem.* **1997**, *18*, 1463–1472, DOI: 10.1002/(sici)1096-987x(199709)18:12<1463::aid-jcc4>3.3.co;2-l.

(5) Bold, B. M.; Sokolov, M.; Maity, S.; Wanko, M.; Dohmen, P. M.; Kranz, J. J.; Kleinekathöfer, U.; Höfener, S.; Elstner, M. Benchmark and Performance of Long-Range Corrected Time-Dependent Density Functional Tight Binding (LC-TD-DFTB) on Rhodopsins and Light-Harvesting Complexes. *Phys. Chem. Chem. Phys.* **2020**, *22*, 10500–10518, DOI: 10.1039/C9CP05753F.

(6) Maity, S.; Daskalakis, V.; Jansen, T. L. C.; Kleinekathöfer, U. Electric Field Susceptibility of Chlorophyll c Leads to Unexpected Excitation Dynamics in the Major Light-Harvesting Complex of Diatoms. *J. Phys. Chem. Lett.* **2024**, *15*, 2499–2510, DOI: 10.1021/acs.jpclett.3c03241.

(7) Maity, S.; Kleinekathöfer, U. Recent Progress in Atomistic Modeling of Light-Harvesting Complexes: A Mini Review. *Photosynth. Res.* **2023**, *156*, 147–162, DOI: 10.1007/s11120-022-00969-w.

(8) Madjet, M. E.; Abdurahman, A.; Renger, T. Intermolecular Coulomb Couplings from Ab Initio Electrostatic Potentials: Application to Optical Transitions of Strongly Coupled Pigments in Photosynthetic Antennae and Reaction Centers. *J. Phys. Chem. B* **2006**, *110*, 17268–81, DOI: 10.1021/jp0615398.

(9) Renger, T.; Müh, F. Theory of Excitonic Couplings in Dielectric Media: Foundation of Poisson-TrEsp Method and Application to Photosystem I Trimers. *Photosynth. Res.* **2012**, *111*, 47–52, DOI: 10.1007/s11120-011-9685-6.

(10) Marin, A.; Doust, A. B.; Scholes, G. D.; Wilk, K. E.; Curmi, P. M. G.; van Stokkum, I. H. M.; van Grondelle, R. Flow of Excitation Energy in the Cryptophyte Light-harvesting Antenna Phycocyanin 645. *Biophys. J.* **2011**, *101*, 1004–1013, DOI: 10.1016/j.bpj.2011.07.012.

(11) Blaustein, M. P.; Kao, J. P. Y.; Matteson, D. R. *Cellular Physiology and Neurophysiology*, 3rd ed.; Elsevier: Philadelphia, PA, 2019.

(12) Maity, S.; Bold, B. M.; Prajapati, J. D.; Sokolov, M.; Kubař, T.; Elstner, M.; Kleinekathöfer, U. DFTB/MM Molecular Dynamics Simulations of the FMO Light-Harvesting Complex. *J. Phys. Chem. Lett.* **2020**, *11*, 8660–8667, DOI: 10.1021/acs.jpclett.0c02526.

(13) Blau, S. M.; Bennett, D. I. G.; Kreisbeck, C.; Scholes, G. D.; Aspuru-Guzik, A. Local Protein Solvation Drives Direct Down-Conversion in Phycobiliprotein PC645 Via Incoherent Vibronic Transport. *Proc. Natl. Acad. Sci. USA* **2018**, *115*, E3342–E3350, DOI: 10.1073/pnas.1800370115.

(14) Dean, J. C.; Mirkovic, T.; Toa, Z. S.; Oblinsky, D. G.; Scholes, G. D. Vibronic Enhancement of Algae Light Harvesting. *Chem* **2016**, *1*, 858–872, DOI: 10.1016/j.chempr.2016.11.002.

(15) Lee, M. K.; Bravaya, K. B.; Coker, D. F. First-Principles Models for Biological Light-Harvesting: Phycobiliprotein Complexes from Cryptophyte Algae. *J. Am. Chem. Soc.* **2017**, *139*, 7803–7814, DOI: 10.1021/jacs.7b01780.

(16) Seibt, J.; Mančal, T. Ultrafast Energy Transfer with Competing Channels: Non-Equilibrium Förster and Modified Redfield Theories. *The Journal of Chemical Physics* **2017**, *146*, DOI: 10.1063/1.4981523.

(17) Meyer-Mölleringhof, M.; Martinez-Azcona, P.; Chenu, A.; Mančal, T. Förster Resonance Energy Transfer with Transient Coherent Effects. 2026; https://arxiv.org/abs/2602.17789.
